# The Adaxial/Abaxial Patterning of Auxin and Auxin Gene in Leaf Veins Functions in Leafy Head Formation of Chinese Cabbage

**DOI:** 10.3389/fpls.2022.918112

**Published:** 2022-06-09

**Authors:** Xiaozhen Yue, Tongbing Su, Xiaoyun Xin, Peirong Li, Weihong Wang, Yangjun Yu, Deshuang Zhang, Xiuyun Zhao, Jiao Wang, Liling Sun, Guihua Jin, Shuancang Yu, Fenglan Zhang

**Affiliations:** ^1^Beijing Vegetable Research Center (BVRC), Beijing Academy of Agriculture and Forestry Science (BAAFS), Beijing, China; ^2^National Engineering Research Center for Vegetables, Beijing, China; ^3^Key Laboratory of Biology and Genetic Improvement of Horticultural Crops (North China), Ministry of Agriculture, Beijing, China; ^4^Beijing Key Laboratory of Vegetable Germplasm Improvement, Beijing, China; ^5^Key Laboratory of the Vegetable Postharvest Treatment of Ministry of Agriculture, Beijing Key Laboratory of Fruits and Vegetable Storage and Processing, Institute of Agri-Food Processing and Nutrition (IAPN), Beijing Academy of Agriculture and Forestry Sciences, Beijing, China

**Keywords:** adaxial/abaxial, auxin, Chinese cabbage (*Brassica rapa* L. ssp. *pekinensis*), leafy head, PIN5

## Abstract

Leaf curling is an essential prerequisite for the formation of leafy heads in Chinese cabbage. However, the part or tissue that determines leaf curvature remains largely unclear. In this study, we first introduced the auxin-responsive marker *DR5::GUS* into the Chinese cabbage genome and visualized its expression during the farming season. We demonstrated that auxin response is adaxially/abaxially distributed in leaf veins. Together with the fact that leaf veins occupy considerable proportions of the Chinese cabbage leaf, we propose that leaf veins play a crucial supporting role as a framework for heading. Then, by combining analyses of QTL mapping and a time-course transcriptome from heading Chinese cabbage and non-heading pak choi during the farming season, we identified the auxin-related gene *BrPIN5* as a strong candidate for leafy head formation. *PIN5* displays an adaxial/abaxial expression pattern in leaf veins, similar to that of *DR5::GUS*, revealing an involvement of *BrPIN5* in leafy head development. The association of *BrPIN5* function with heading was further confirmed by its haplo-specificity to heading individuals in both a natural population and two segregating populations. We thus conclude that the adaxial/abaxial patterning of auxin and auxin genes in leaf veins functions in the formation of the leafy head in Chinese cabbage.

## Introduction

Chinese cabbage (*Brassica rapa* L. ssp. *pekinensis*) is of great importance to vegetable supply in Asia, where about 70% of the world’s output is grown. The leafy head is the edible part of Chinese cabbage, and the timing and tightness of the head are crucial to its production and commercial quality. Throughout the growing season, Chinese cabbage plants undergo four typical developmental steps that include the seedling stage, rosette stage, folding stage, and heading stage (Chen, 1984; Zhang et al., 2022). Among these, the late rosette stage at which the plant leaves begin to curl is pivotal for the formation of a leafy head (Mao et al., 2014). Leafy head formation depends on both external environmental elements, such as temperature, water, and light intensity, and intrinsic genetic factors, including auxin dynamics and the carbon-to-nitrogen ratio. Among these aspects, auxin is stated to be the most critical, and an asymmetrical distribution of auxin in the leaves of Chinese cabbage can determine the fate of head formation (Ito and Kato, 1957; He et al., 2000; Yu et al., 2000). However, to the best of our knowledge, the distribution pattern of auxin in leaves during leafy head formation of Chinese cabbage has not been visualized, and the molecular mechanism and genetics that underlie leafy head development remain poorly understood.

Leaf curling is an indispensible prerequisite for the development of leafy heads in Chinese cabbage. However, the particular part or tissue of the leaf that determines or is the dominant factor in controlling the curvature remains largely unclear. Previous studies have focused on the leaf tips and margins in which auxin signaling or other relevant factors are relatively easy to study. As an important component of the leaf, leaf veins are netted distributed throughout the leaf; they not only transport nutrients to the leaf, but also play a crucial role as a structural framework. As known to all, Chinese cabbage has a dramatically higher proportion of leaf veins in the whole leaf comparing with its ancestor Arabidopsis, and the proportion is also significantly higher than that of pak choi, another leafy *B. rapa* crop that shares the same AA genome with Chinese cabbage. Intriguingly, we noticed that the proportion increases rapidly during the folding and heading stages of Chinese cabbage (Supplementary Figure S1). Subsequently, during the formation of the leafy head of Chinese cabbage, both the primary and secondary veins start to bend, and a large leaf midrib forms. We thus hypothesize that leaf veins play an untapped role in leaf curling in Chinese cabbage.

During curling, the leaf is shaped by the asymmetrical growth of cells. In Arabidopsis, proper establishment of the adaxial/abaxial (hereafter abbreviate to Ad/Ab) axis of polarity within organ primordia is a necessary condition for asymmetric growth and development (Bowman et al., 2002). Auxin is one of the plant hormones involved in regulating polar growth (Qi et al., 2014). The auxin response factors ARF3 and ARF4 act together with KANs (KANADI proteins) to promote abaxial fate in leaf initiation (Pekker et al., 2005), and the *arf3arf4* double mutant shows an obvious curling leaf phenotype due to the loss of abaxial axis side polarity (Guan et al., 2017). It has been reported that the dynamic balance of Ad/Ab polarity leads to leafy head formation in Chinese cabbage based on genetic and transcriptome results (Liang et al., 2016; Gu et al., 2017); additionally, several auxin signaling genes are reported to participate in leafy head formation, such as *BrPIN1*, *BrLAX1* and *BrSAURs* (Gao et al., 2017; Gu et al., 2017; Li et al., 2019b). All of these studies unequivocally demonstrated the role of auxin genes in the establishment of leaf margin Ad/Ab polarity at the molecular level (Cheng et al., 2016; Liang et al., 2016). However, although the importance of auxin in leafy head formation has been widely reported, we noticed that there has been no use of a visible marker, such as *DR5::GUS*, to accurately track the dynamic distribution patterns of auxin signaling during the process of leaf development in Chinese cabbage.

In order to characterize the role of auxin in leaf curling and determining leafy head formation during the growth of Chinese cabbage, we observed the expression pattern of the auxin-responsive *DR5::GUS* construct during the four typical developmental stages of Chinese cabbage. We found that the GUS signal in the whole leaf initially gets stronger and then weakens gradually as the plant grows. Moreover, we observed intense GUS signals, distributed adaxially/abaxially, in the exact areas of the leaf veins where the Chinese cabbage leaf starts to curve. Furthermore, by combining the results of bulked segregant analysis (BSA), quantitative trait locus (QTL) mapping, and time-course RNA-seq data, we found that the auxin polar transport protein PIN5 and auxin response gene *SAUR21* are active factors in the formation of the leafy head, which was further confirmed by haplotype analysis in two segregating populations and a natural population. *PIN5* and *SAUR21* mainly express and display Ad/Ab distribution patterns in leaf veins. Thus, in this study we have demonstrated a previously overlooked role of auxin and auxin genes of leaf veins in the development of leafy head formation in Chinese cabbage.

## Results

### Auxin Is Dynamically Distributed in the Chinese Cabbage Leaf During Growth

**Figure 1:**
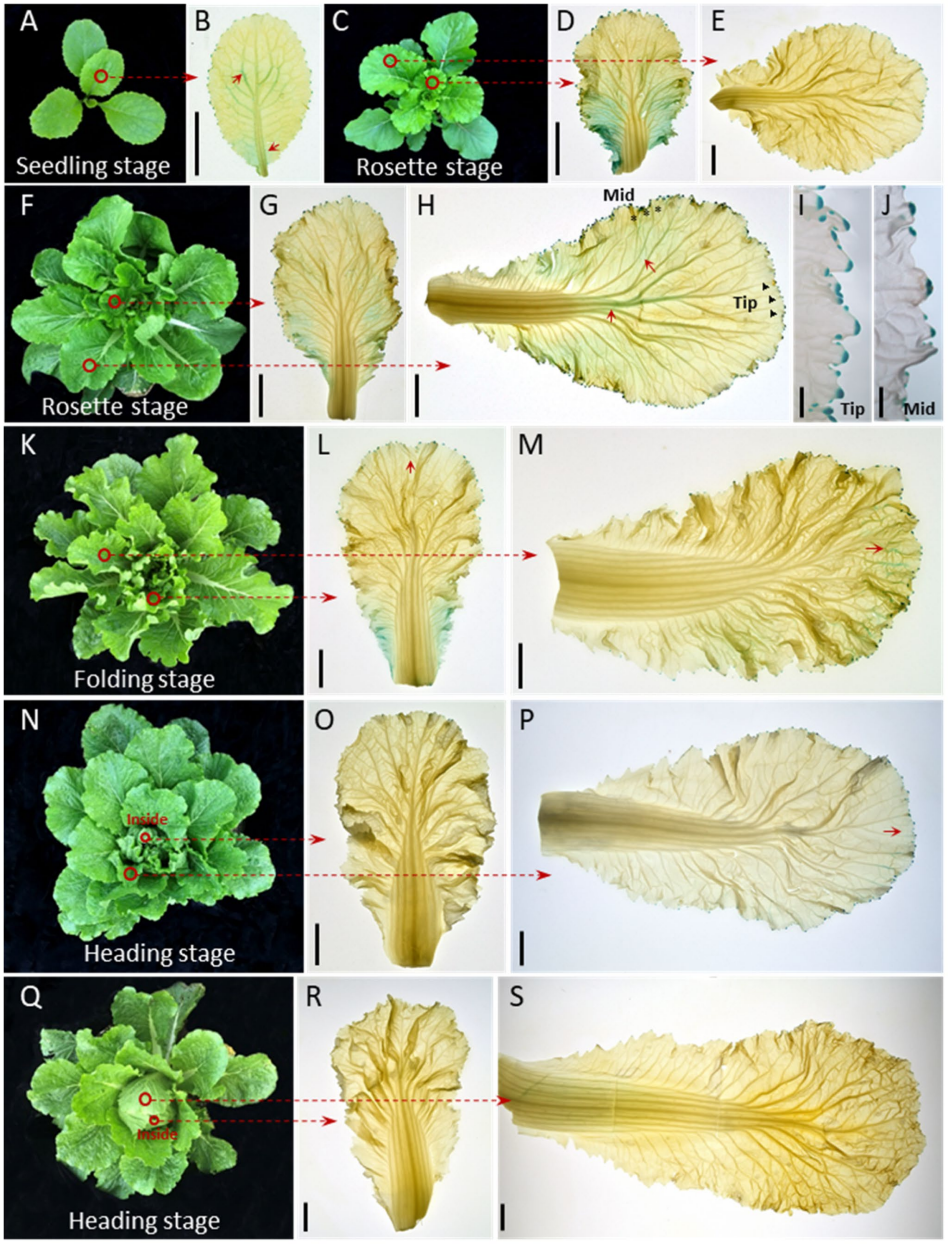
Auxin is dynamically distributed in the Chinese cabbage leaf during growth. Expression pattern of *DR5::GUS* in seedling (28 days old) **(A)**, rosette (44 and 50 days old, respectively) **(C–J)**, folding (57 days old) **(K–M)** and heading (67 and 87 days old, respectively) stage leaves **(N–S)**. The 1st-2nd innermost leaves **(B**,**D**,**G**,**L**,**O**,**R**) and the 3rd-5th outermost leaves of the plants were harvested for GUS staining **(E**,**H**,**M**,**P**,**S)**. For inner leaves, the *DR5::GUS* signals were expressed over almost the entire leaf at the seedling and rosette stages **(B,D,G)**, then gradually decreased **(L,O,R)**. For the outer leaves, the GUS signals were visible around the whole leaf edge **(E,H)** and converge on the leaf apex especially after the leafy head formed (**P**, red arrows). The GUS signals in leaf veins shifted from the middle to the apical part of the leaf **(H**,**M)** and show a net-like pattern prior to leaf curling **(H)**. **(I,J)** Show magnified vies of the leaf tip (black triangle) and mid-margin (abbreviated to Mid, black asterisk) from **(H)**. Red circles indicate the leaves used for GUS staining. The short red arrows show the areas with strong GUS staining signals. Scale bars: 2 cm **(B,D,E,G,H,L,M,O,P,R,S)**, 2 mm **(I,J)**.

The auxin response reporter *DR5::GUS* is used to visualize the auxin distribution pattern in plants (Ulmasov et al., 1997; Sabatini et al., 1999). Transgenic Chinese cabbage plants of *DR5::GUS* at the seedling (28 days old), rosette (44 and 50 days old), folding (57 days old), and heading (67 and 87 days old) stages were used in the study. GUS staining of young leaves showed that, in seedlings (Figure 1A), auxin signaling was present in the whole leaf blade, with slightly stronger signals at the bottoms, margins, and veins of the leaf (Figure 1B). As the plants grew (Figures 1C,F), the GUS signal intensity increased, especially in the lower parts of the leaves, including the margins and veins (Figures 1D,G). The GUS signals in the midrib and primary leaf veins and the leaf margins were extremely clear in the late rosette stage when the leaves were about to curl (Figure 1H, red arrows; Figures 1I,J). In the folding stage (Figure 1K), we noticed that the auxin signals shifted to the leaf apexes and margins, with clear signs of movement through the leaf veins (Figures 1L,M, red arrows). Finally, after the formation of the leafy head (Figures 1N,Q), the GUS signals gradually weakened or even disappeared (Figures 1O,P,R,S). In general, our GUS staining showed a dynamic auxin signaling pattern, both spatially and temporally, in the Chinese cabbage leaf: the *DR5::GUS* signals first become stronger and then weaken over the whole growing season, and the auxin signal shifts from the bottom to the top or margins of the leaf.

### Epidermal Cells of Curling Leaf Veins Show an Asymmetrical Growth Pattern

**Figure 2:**
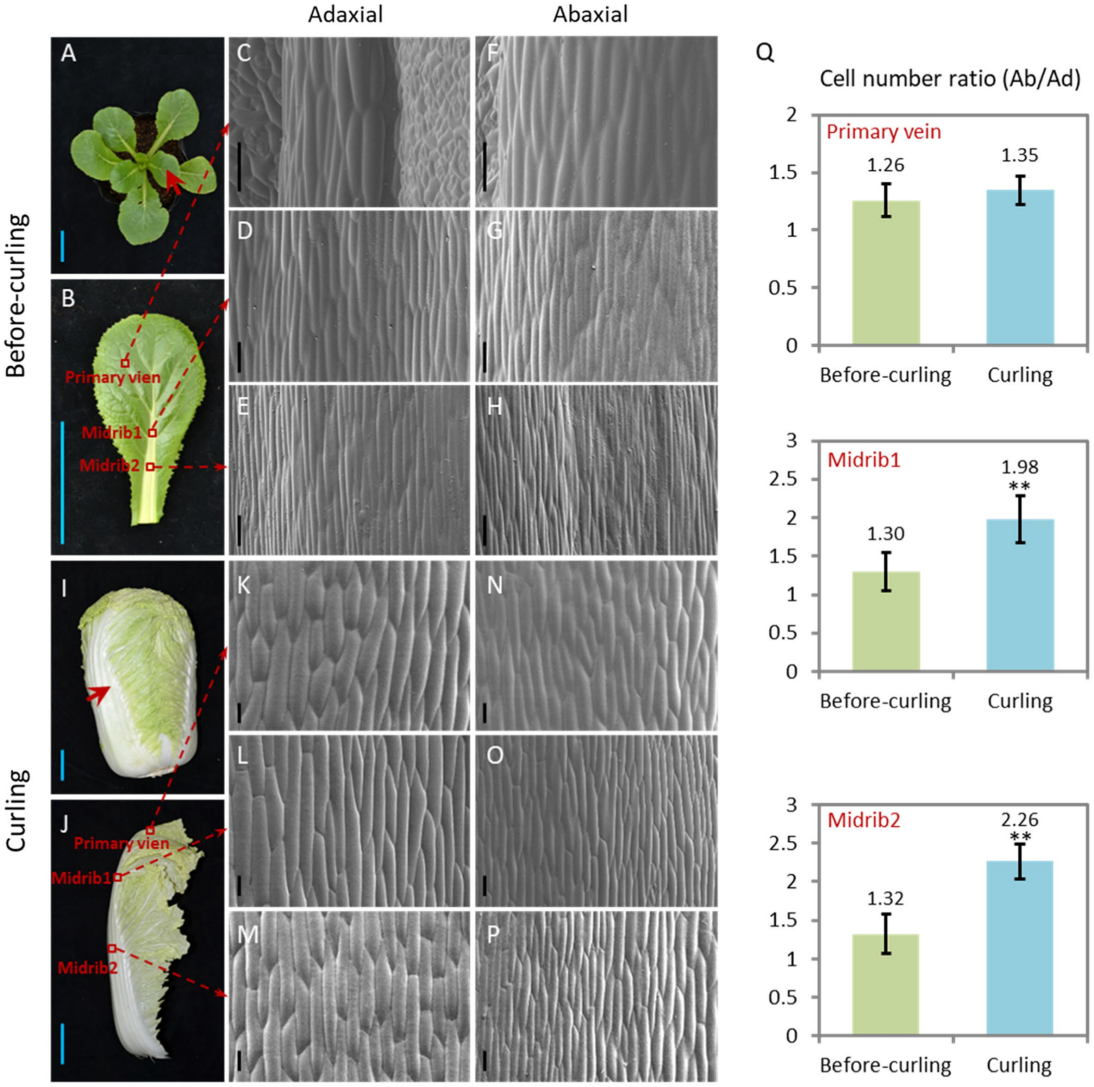
Epidermal cells of the curling veins show an asymmetrical growth pattern. Morphological observation of the adaxial/abaxial (abbreviated to Ad/Ab) epidermal cells of the leaf veins in before-curling **(A–H)** and curling **(I–P)** plants. **(C/F,D/G,E/H)** Scanning electron micrographs of the Ad/Ab cells of the primary vein, higher midrib part (midrib 1), and lower midrib part (midrib 2), respectively, in before-curling plants. **(K/N,L/O,M/P)** Scanning electron micrographs of the Ad/Ab cells of the above corresponding vein parts in curling plants. **(Q)** The cell number ratios (Ab/Ad) of leaf vein epidermis. Red circles and arrows indicate the leaf vein areas used for photographing and cell number calculation; Ad/Ab, adaxial/abaxial; Values are means ± SD (*n* = 6–23). Asterisks indicate significant differences using Student’s *t*-test ^**^p < 0.01. Scale bars: 5 cm **(A**,**B**,**I**,**J)**, 100 μm **(C**–**H**,**K**–**P)**.

We focused our study on leaf veins because of their untapped supportive function and their increasing proportion (Supplementary Figure S1) of the whole leaf during leafy head formation. Morphological features of the Ad/Ab epidermal cells in the leaf veins before and during curling were characterized (Figure 2). In terms of cell size, both adaxial and abaxial epidermal cells were larger in the two evaluated parts of the leaf veins, including the midribs and primary leaf veins, from the before-curling to the curling stage, and the adaxial epidermal cells were slightly larger than the abaxial epidermal cells in both stages (Figures 2C–H,K). With regard to cell number increase, the abaxial side divided much quicker than the adaxial side, and dramatic increases in the cell number ratio (Ab/Ad) were therefore found in all three evaluated parts in the transition period from before-curling to curling (Figure 2Q; Supplementary Figure S2). All of these results demonstrate that the epidermal cells of leaf veins showed an Ad/Ab growing pattern in curling leaves.

### Auxin Signaling Is Distributed Abaxially/Adaxially in Leaf Veins

**Figure 3:**
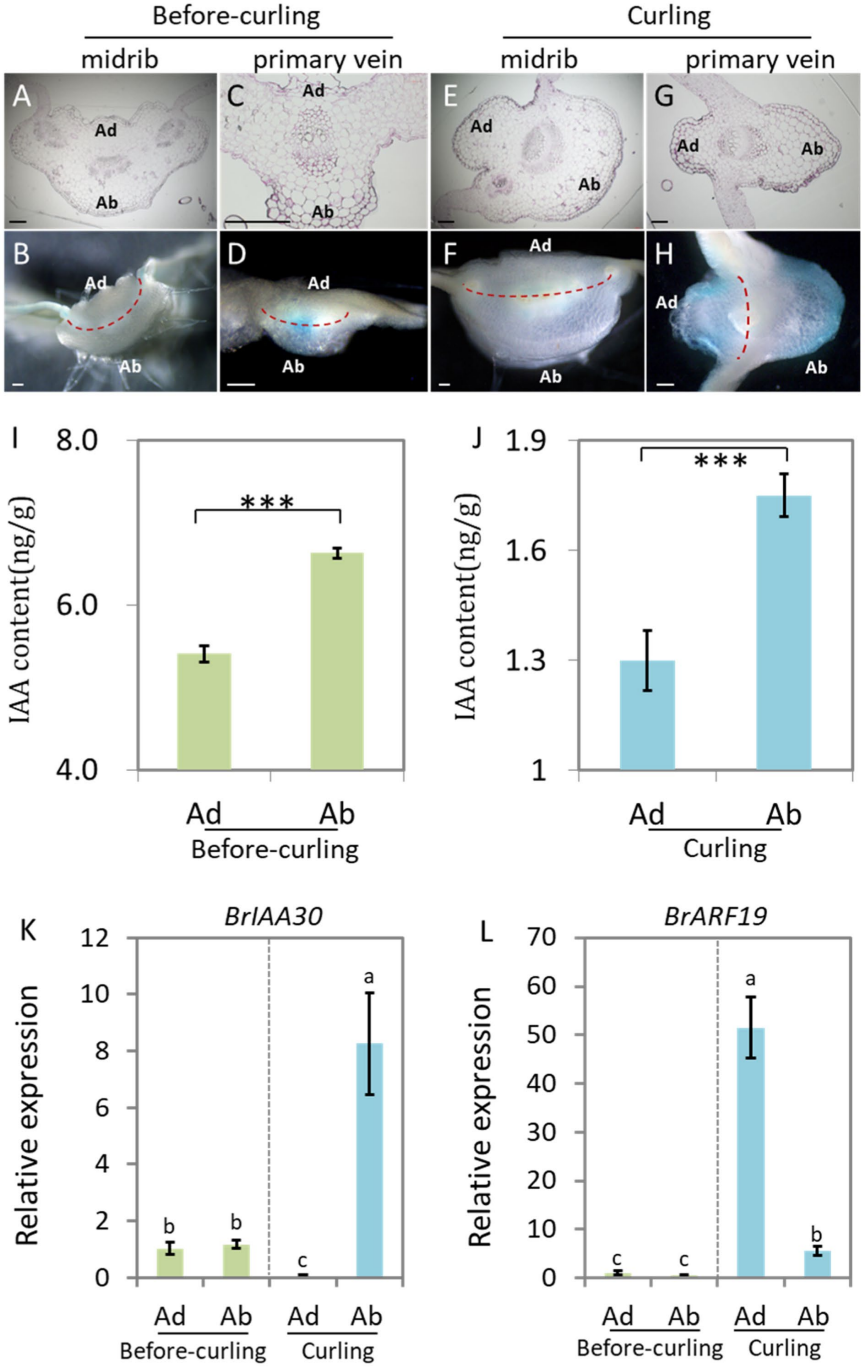
Auxin shows abaxial/adaxial distribution in the leaf veins. Transverse sections of GUS stained leaf veins in before-curling plants from Figure 1B **(A–D)** and curling plants from Figure 1H **(E**–**H)**. **(A,B,E**,**F)** Midribs; **(C**,**D**,**G**,**H)** primary veins. **(A**,**C**,**E**,**G)** Ultrathin sections; **(B**,**D**,**F**,**H)** free-hand sections. **(I,J)** Measurement of endogenous IAA in midribs of the before-curling and curling leaf veins of which developmental stages were displayed in Figures 1B,H. **(K,L)** Expressions of *BrIAA30* and *BrARF19* in midribs of before-curling and curling plants, respectively. Error bars represent the standard errors derived from three replicates. Asterisks indicate significant differences using Student’s *t*-test ^***^p < 0.01. Different lower case letters represent highly significant differences by Student’ s *t*-test, *p* ≤ 0.01. The materials **(I–L)** used for analysis were taken from the leaf parts shown in Supplementary Figures S3E,F. Scale bars: 200 μm. Ad/Ab, adaxial/abaxial.

Auxin is reported to be the key hormone in the regulation of the Ad/Ab cell polar growth in plants (Manuela and Xu, 2020). In order to test whether auxin signaling is distributed adaxially/abaxially in leaf veins of Chinese cabbage, we first performed semi-thin sectioning of the *DR5::GUS* stained leaf veins at the before-curling and curling stages shown in Figures 1B,H, respectively. However, due to the limited thickness, we were unable to photograph the GUS signal in transverse sections of the leaf veins (Figures 3A,C,E,G).

We therefore used manual sections of both before-curling (Figures 3B,D) and curling (Figures 3F,H) leaf veins for further study. No visible asymmetrical distribution pattern of DR5::GUS was observed in the before-curling leaf veins; however, asymmetrical Ad/Ab distribution pattern was found in the primary leaf veins of the curling leaves (Figures 3D,H). Furthermore, we observed that *DR5::GUS* was mainly expressed in the vascular bundles before curling, while at curling stage, the auxin was mainly found in the epidermal and mesophyll cells of the leaf veins and was distributed adaxially/abaxially; and the distribution area of the *DR5::GUS* signals were larger on the abaxial side than on the adaxial side in the curling leaves. Moreover, we found that auxin was continuously distributed in the epidermal cells of the abaxial sides of the primary leaf veins, but *DR5::GUS* was expressed noncontiguous on the adaxial side.

DR5::GUS is an indicator of the intensity of auxin signaling. To further evaluate if auxin concentration is different between the adaxial and abaxial cells, we then detected the auxin contents of both sides of the epidermal cells in leaf veins (Supplementary Figure S3F) at before-curling and curling stages to further display its distribution pattern. We found that the IAA content in the abaxial cells was significantly higher than that of adaxial cells at the two tested stages; and the increasing tendency, not the net content, of IAA in the abaxial vein cells was more dramatic in the curling leaves (Figures 3I,J).

To further confirm the Ad/Ab distribution pattern of auxin in Chinese cabbage leaf veins, we next evaluated the expression of *BrIAA30* (*BraA07g024980.3C*) and *BrARF19* (*BraA08g028300.3C*), homologs of which have been used as marker genes to indicate whether auxin shows an Ad/Ab distribution pattern in leaves of Arabidopsis, in the primary leaf veins (Li et al., 2006; Christie et al., 2011; Liu et al., 2018). The results showed that there were no differences in the expression of *BrIAA30* and *BrARF19* in the abaxial and adaxial cells before leaf curling; however, significant expression differences were indeed noted at the curling stage, which was in line with the auxin distribution pattern observed in the midribs (Figures 3K,L). In addition, the expression of *BrIAA30* and *BrARF19* was also tested in the marginal tissues of the leaf tips and middle edges which showed strong *DR5::GUS* signals in the same samples used in Figures 1I,J and Supplementary Figure S3E. In this case, however, only slight, if any, differences in auxin response were found in both the before-curling and curling stages (Supplementary Figure S4). Therefore, the measurement of auxin, observation of DR5::GUS signal, and the expression analysis of auxin marker genes provide strong evidences that auxin is adaxially/abaxially distributed in leaf veins.

### Interruption of Auxin Transport Leads to Asymmetrical Growth of Abaxial/Adaxial Epidermal Cells of Leaf Veins and Consequent Leaf Curling at Seedling Stage

**Figure 4:**
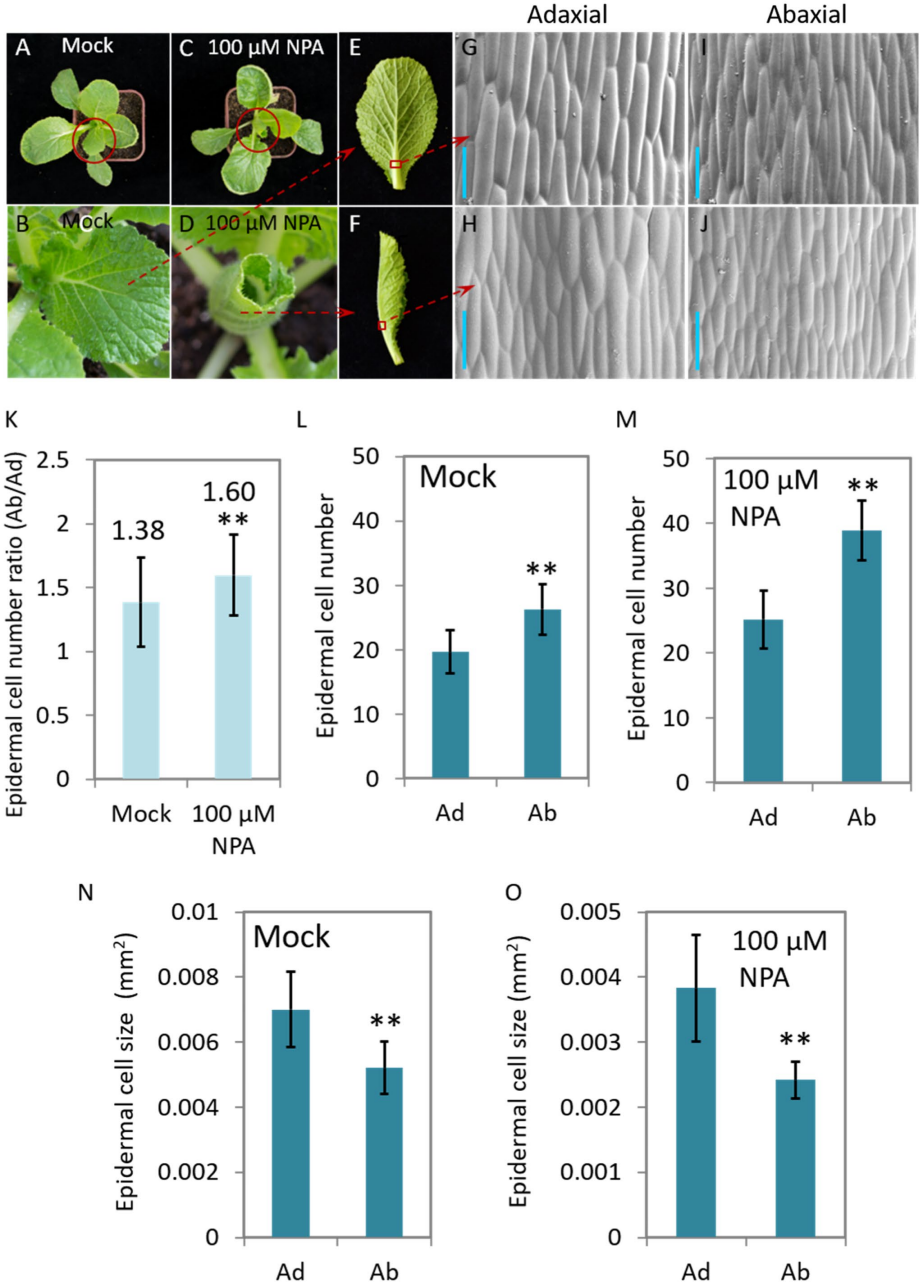
The NPA-mediated interruption of auxin transport leads to leaf curling and asymmetrical growth of abaxial/adaxial vein epidermal cells at seedling stage. Phenotypes of the plants (40-day-old) treated with 100 μM NPA **(C**,**D**,**F)** and mock **(A**,**B**,**E)**. **(B,D)** Magnified pictures of the leaves marked with red circles in **(A,C)**; **(G–J)** scanning electron micrographs of the Ab/Ad cells of the midrib parts indicated by the red squares in **(E,F)**. **(K)** The cell number ratios (Ab/Ad) of leaf vein epidermis. **(L,M)** Adaxial and abaxial epidermal cell numbers of the midrib parts displayed in **(G–J)**. Cell numbers in areas of 0.134 mm^2^ (Mock) and 0.093 mm^2^ (treated with 100 μM NPA), respectively, were counted. **(N,O)** Mean size of the adaxial and abaxial epidermal cells in the midrib parts of the leaf veins displayed in **(G–J)**. Values are means ± SD (*n* = 32–48). Asterisks indicate significant differences using Student’s *t*-test ^**^p < 0.01. Scale bars: 100 μm **(G**–**J)**. Ad/Ab, adaxial/abaxial.

N-1-naphthylphthalamic acid (NPA) is an auxin efflux inhibitor that targets on the auxin transporter PIN proteins and consequently interrupt the distribution of auxin. We therefore treated 40-day-old Chinese cabbage seedlings with NPA (100 μM) to see the morphological change and auxin response of the plants. We found that the leaves curled inwardly after 7 days of treatment, a morphology which is very similar to that of curling leaves of heading Chinese cabbage (Figure 2J). We then observed the growth, including cell number and cell size, of both abaxial and adaxial epidermal cells of leaf veins. We found that the number of adaxial cells remained stable after NPA treatment, while the number speedily increased for the abaxial cells, expressing as that the cell number ratios (Ad/Ab) increased from 1.38 to 1.60 (Figures 4K–M). For cell size, NPA treatment led to reduced expansion of both abaxial and adaxial epidermal cells to a similar degree (Figures 4N,O).

### Identification of Auxin Genes Expressed Adaxially/Abaxially in Leaf Veins During Leafy Head Formation

**Figure 5:**
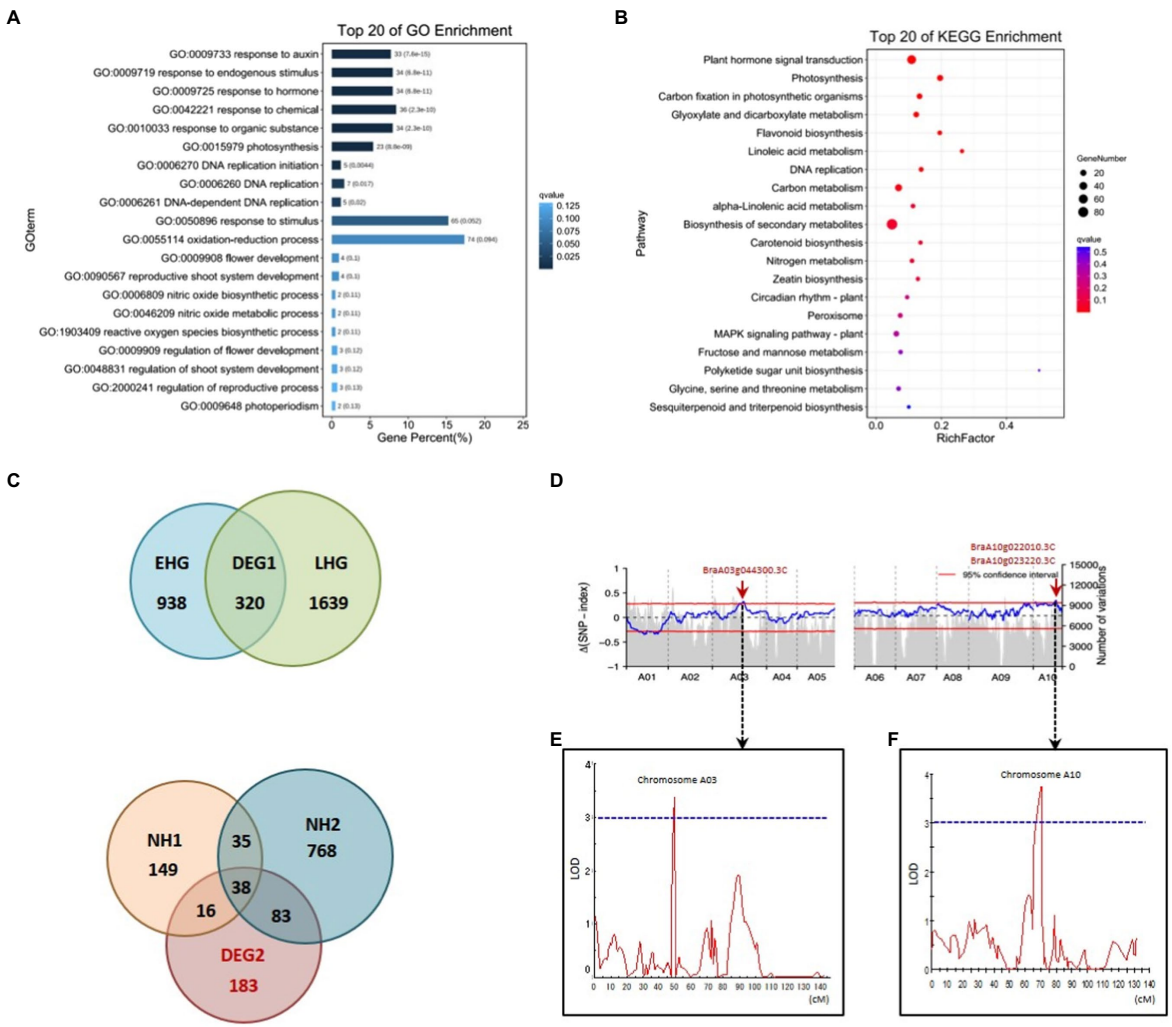
Coupling of RNA-seq, BSA, and QTL mapping to identify auxin genes functioning in leafy head formation. GO **(A)** and KEGG pathway **(B)** analysis of DEGs from the RNA-seq data of Ab/Ad tissue of curling leaf veins (see materials and analysis flow chart in Supplementary Figures S3D,F, S5A). **(C)** Venn diagrams for the DEGs of different comparisons of different *Brassica rapa* crops during the farming season (see materials and analysis flow chart in Supplementary Figures S3E, S5E). EHG, the DEGs of early-heading Chinese cabbage; LHG, the DEGs of late-heading Chinese cabbage; NH, the DEGs of the non-heading pak choi materials. **(D)** The *Δ*SNP index values (95% confidence interval) used for the association analysis of heading. **(E,F)** Overlapped intervals identified by QTL mapping. The vertical axis indicates LOD-scores; The horizontal axis shows genetic positions; The LOD-score threshold of 3 is indicated by the horizontal dashed line. DEG, differentially expressed gene; GO, Gene Ontology; KEGG, Kyoto Encyclopedia of Genes and Genomes; BSA, bulk segregant analysis; LOD, Logarithm of odds. Ad/Ab, adaxial/abaxial.

In order to identify genes that show Ad/Ab expression in leaf veins that were also differentially expressed during Chinese cabbage leaf folding, we chose both the abaxial and adaxial surface cells in the same position on both sides of the midrib at the before-curling and curling stages for transcriptome analysis (Supplementary Figures S3B,D,F). A total of 606 and 1,068 DEGs were found to be expressed adaxially/abaxially in the before-curling and curling leaves, respectively. Gene Ontology (GO) analysis showed that the top five enriched GO terms in the “biological process” category for the 606 DEGs in the before-curling leaves were “oxidation–reduction,” “sulfate transmembrane transport,” “single-organism metabolic,” “carbohydrate metabolic,” and “lipid transport “(Supplementary Figure S5B), while the top five “biological process” GO terms for the 1,068 DEGs in the curling stage leaves were “response to auxin,” “response to endogenous stimulus,” “response to hormone,” “response to chemical,” and “response to organic substance” (Supplementary Figure S5A; Figure 5A). KEGG analysis further showed that the DEGs in the curling leaf veins were enriched in “plant hormone signal transduction” (Figure 5B), while the DEGs in the before-curling leaves were mainly enriched in “biosynthesis of secondary metabolites” (Supplementary Figure S5C). In summary, the DEGs in the curling, but not before-curling, leaves are more likely to be involved in multiple auxin signaling pathways, including auxin response, signaling transduction, polar transport, and biosynthesis pathways (Supplementary Table S2).

To further narrow down the list of genes involved in leafy head formation, we conducted transcriptome sequencing of a time-course of samples taken from different *B. rapa* subspecies during the farming season, including six Chinese cabbage lines and three non-heading pak choi lines. A total of 183 genes that were specific to the leafy head formation process in Chinese cabbage were found to be differentially expressed (Figure 5C; also see detailed analytical strategy in Supplementary Figure S5E). We then found that the auxin genes *BraA10g022010.3C* (*SAUR21*), *BraA08g015020.3C* (*SAUR34*), *BraA07g039050.3C* (*WAT1*), *BraA03g007900.3C* (*RVE1*), and *BraA06g023540.3C* (*PHOT1*) were among the overlapping genes of the 1,068 and 183 DEGs which were specific to the curling stage and leafy head formation, respectively (Supplementary Table S3; Redman et al., 2004; Rawat et al., 2009; Christie et al., 2011; Spartz et al., 2012; Ranocha et al., 2013).

We then used two segregating populations, including an F_2_ and a double haploid (DH) population generated from the same F_1_ plants of Chinese cabbage × turnip (Su et al., 2021), to identify genes that are associated with heading. We first performed BSA in the F_2_ population consisting of 1,565 individuals to identify heading loci. DNA bulks of the heading- and non-heading-progeny, 111 and 81 individuals, respectively, were used for DNA sequencing, SNP calling, and consequent *Δ*(SNP index) plotting. We found highly contrasting patterns in the SNP-index graph for the heading- and non-heading bulks in eight regions (bQTL, BSA-QTL; Supplementary Tables S4, S5). Among the eight regions, the intervals A10:15,447,261-16,459,616 and A03:20,480,010-22,959,446 were also found to overlap with the candidate loci identified by QTL mapping in the DH population. The candidate gene list was then finally narrowed down to only *BrSAUR21* and *BrPIN5* by combining all of the transcriptome, BSA, and QTL mapping data.

### *BrSAUR21* and *BrPIN5* Are Expressed Adaxially/Abaxially in Leaf Vein

**Figure 6:**
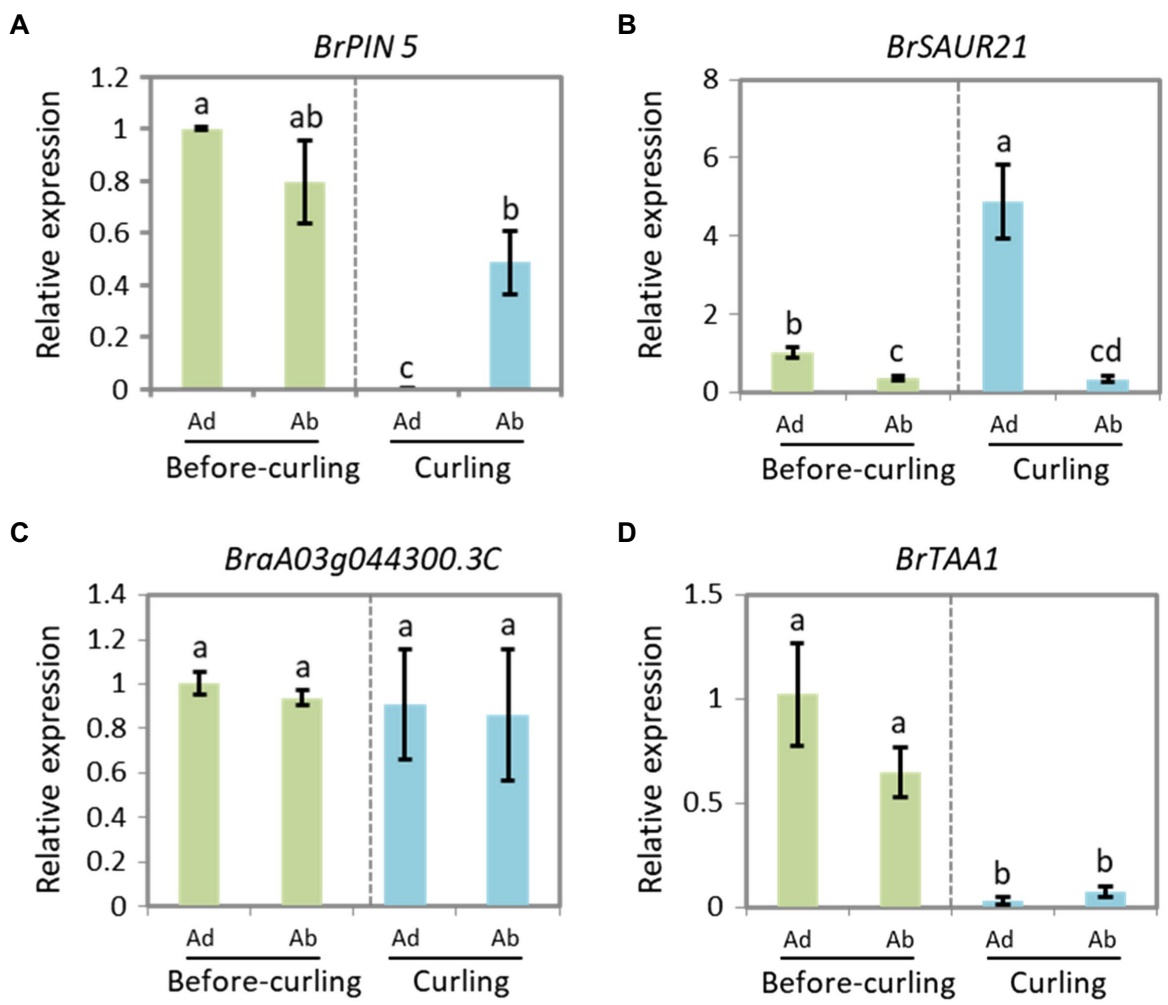
Expressions of BrPIN5 **(A)** and BrSAUR21 **(B)**, as well as BraA03g044300.3C **(C)** and BrTAA1 **(D)** in abaxial and adaxial midribs of before-curling and curling leaves.

To further clarify the role of *BrSAUR21* and *BrPIN5* in the process of leafy head formation, we evaluated the expression patterns of *BrSAUR21* and *BrPIN5* in the midribs, leaf tips, and margins. *BrTAA1* (*BraA02g019880.3C*), which is pivotal to auxin biosynthesis but was not identified as a candidate gene from the transcriptome analysis, BSA, and QTL mapping data, and *BraA03g044300.3C* (*AT5G61290*), which is annotated as an auxin gene in the DEG list but is not located in any of the QTL regions (Supplementary Table S3), were used as negative controls. For *BrPIN5*, clear Ad/Ab expression pattern was only detected in curling leaves, but not in before-curling leaves (Figure 6A); for *BrSAUR21*, Ad/Ab expression pattern were found in both the before-curling and curling leaf veins, and the asymmetric pattern was strengthened in the curling leaves (Figure 6B). Both of the two negative control genes showed no Ad/Ab expression pattern (Figures 6C,D). We also examined the expression of the four genes in the leaf tips and mid-margins. The expression of *BrPIN5* was higher in the leaf tips than in the margins in both before-curling and curling leaves, while an inverse expression pattern was found for *BrSAUR21* in curling leaves (Supplementary Figures S6A,B). No difference in expression was found for *BraA03g044300.3C* (*AT5G61290*) in leaf tips and margins in both before-curling and curling leaves, and *BrTAA1* expression was slightly higher in leaf tips and mid-margins in leaves at both stages (Supplementary Figures S6C,D).

To clarify if the Ad/Ab expression pattern of *BrSAUR21* and *BrPIN5* is specific to the heading *B. rapa* crop, we then investigated their expressions in a non-heading pak choi line. *BrPIN5*, not *BrSAUR21*, displayed Ad/Ab expression patterns in pak choi, but to a less extent comparing with that of Chinese cabbage (Figure 6 and Supplementary Figure S7). Besides, cell expansion of Ad/Ab epidermis in the pak choi line was also checked. A mild quicker division of the abaxial epidermal cells was found in pak choi (Supplementary Figure S8), similar to that of Chinese cabbage (Figure 2). However, the cell number ratios (Ab/Ad, 1.17, Supplementary Figure S8F) was much lower than that of Chinese cabbage (1.98, Figure 2Q), suggesting that the abaxial epidermal cells of leaf veins of Chinese cabbage divide at a higher rate than pak choi, which may finally lead to leaf curling in Chinese cabbage but not in pak choi. These results add further evidence that the expression pattern of auxin and auxin genes play crucial roles in cell growth and consequent leaf curling.

### *BrSAUR21* and *BrPIN5* Are Key Auxin Genes in Leafy Head Formation

**Figure 7:**
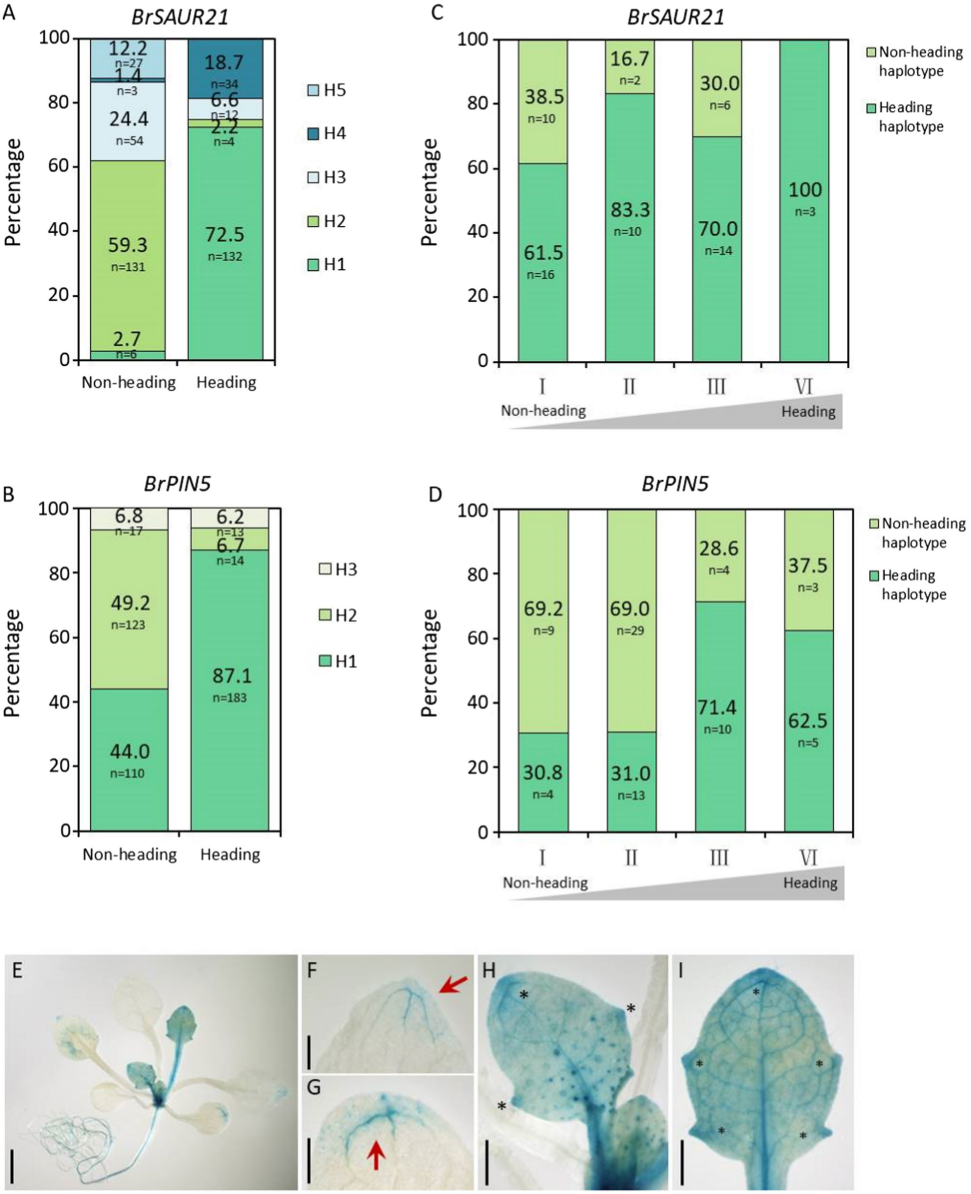
Haplotype analysis of *BrSAUR21* and *BrPIN5* in natural lines and in two DH segregation populations. Haplotype analysis of *BrSAUR21* **(A)** and *BrPIN5* **(B)** based on the SNPs and Indels in the gene bodies using 460 natural lines (see SNPs/Indels of *BrSAUR21* and *BrPIN5* in Supplementary Figure S9. The definitions of haplotypes H1-H5 for *BrSAUR21* **(A)** and H1–H3 for *BrPIN5* **(B)** were denoted in details in Supplementary Figure S9. Haplotype analyses of *BrSAUR21* **(C)** and *BrPIN5* **(D)** using the two DH populations (see details in “Materials and Methods”). For both genes, the heading haplotype was defined as the haplotype that is from Chinese cabbage; I to IV indicate the heading levels from non-heading to heading. **(E–I)** The *PIN5::GUS* expression pattern in leaf veins of Arabidopsis. Red arrows and black asterisks show the regions with strong signals in cotyledon **(G)** and true leaves **(F**,**H,I)**. Scale bars: 2 mm **(E)** 500 μm **(F–I)**.

To further determine whether *BrSAUR21* and *BrPIN5* participate in heading, we accessed genomic data from a total of 460 *B. rapa* lines, consisting of 210 Chinese cabbage (ssp. *pekinensis*), 250 pak choi (ssp. *chinensis*, ssp. *chinensis* var. *wu-tsai* Lin., ssp. *chinensis* var. *narinosa* Lin., ssp. *chinensis* var. *parachinensis* Lin.), 66 turnip (ssp. *rapifera*), and nine ssp. *oleifera* accessions by downloading the genomic data from public resources (Cheng et al., 2016; Su et al., 2018, 2021). All 210 Chinese cabbage accessions were classified as belonging to the heading group, while all of the 250 pak choi lines were classified as non-heading group. We conducted gene haplotype analyses of *BrSAUR21* and *BrPIN5* based on the SNPs and Indels in the gene bodies with the promoter regions included (2 kb upstream of the start codon) in the two groups. A total of five and three haplotypes were found for *BrSAUR21* and *BrPIN5*, respectively. We found that Haplotypes 1 for both genes (designed as *BrSAUR21*^H1/H1^ (*n* = 132) and *BrPIN5* ^H1/H1^ (*n* = 183)) were found to represent the largest clusters in the heading group, and their frequencies were 72.5% in the heading group and 2.7% in the non-heading group, and 87.1% in the heading group and 44% in the non-heading group, respectively, suggesting that both *BrSAUR21* and *BrPIN5* probably experienced strong selection during Chinese cabbage formation (Figures 7A,B; Supplementary Figure S9).

To further determine the genetic role of *BrSAUR21* and *PIN5* in leafy head formation, we conducted haplotype analyses in two DH populations, DH80 and DH85, generated from the F_1_ plants of Chinese cabbage × turnip (non-heading type) and Chinese cabbage × pak choi (Su et al., 2021), respectively. Both the plant phenotypes and the genotypes of *BrSAUR21* and *PIN5* of the two populations were then collected and used for analysis. The frequency of the heading haplotypic *BrPIN5* increased from 76.9% in the non-heading lines to 88.9% (*n* = 45) in the heading lines in the DH80 population, and from 30.8 to 62.5% (*n* = 32) in the DH85 population; while the frequency of the heading haplotypic *BrSAUR21* increased from 61.5 to 100% (*n* = 43) in the DH80 population (Figures 7C,D and Supplementary Figure S10). There is no haplotypic difference of *BrSAUR21* in the DH85 population.

### *PIN5* Is Expressed in the Leaf Veins

From the RNA-seq data, we noticed that the expression of *BrPIN5* was significantly higher in leaf veins than in the mesophyll. To test whether *BrPIN5* is involved in leaf vein patterning, *AtPIN5::GUS* transgenic plants were generated (Le et al., 2014) and used for GUS staining analysis. We found that the *PIN5::GUS* signal was ubiquitously expressed in young true leaves at the seedling stage, and stronger signals were observed in the leaf vein tips and teeth (Figure 7E). Intriguingly, but as expected, the strongest GUS signals were expressed in the leaf midribs (Figures 7H,I). These results further show the involvement of auxin and auxin genes in leaf vein patterning.

## Discussion

Auxin regulates plant development in general, and has been reported to participate in leafy head formation in Chinese cabbage (Li et al., 2019b). Previous studies have suggested that the uneven distribution of auxin, especially its distribution pattern at the leaf margin, affects the formation of leafy heads (Gao et al., 2017). As an integral part of the leaf, leaf veins transport water, nutrients, and energy to the rest of the leaf while also providing structure, support, and growth direction to plant leaves. Compared with *A. thaliana*, the leaf veins of Chinese cabbage, including the leaf midrib and primary/secondary veins, occupy a large proportion of the whole leaf, and the proportion further increases at the late-heading stage (Supplementary Figure S1). This unique morphological feature suggests a potentially untapped function of leaf veins in Chinese cabbage, as well as in other heading leafy crops. Here, we performed a comprehensive analysis of the expression pattern of auxin in Chinese cabbage leaves (Figure 1), especially the leaf veins, over an entire farming season, and found dynamic expression patterns in both leaf veins and leaves. It is worth noting that we found a strong Ad/Ab distribution of auxin in Chinese cabbage leaf veins.

Auxin controls leaf and vein development (Scarpella et al., 2010). Leaf initiation is caused by high intracellular auxin concentration at the margins that result from directional auxin transport, mainly mediated by *PIN1*, toward the convergence point (Scarpella et al., 2006). We clearly observed strong *DR5::GUS* signals at the convergence points of the leaf margins in Chinese cabbage (Figures 1I,J), which is in accordance with the observations in Arabidopsis. A leaf is the combined results of both leaf margin and vein development. In Arabidopsis, primary leaf morphogenesis temporally coincides with the formation of the major veins (i.e., the midvein and lateral veins), and a suite of mutants exist in which leaf shape and vascular pattern defects are coupled (Liu et al., 2018). Although the interdependency between leaf form acquisition and vascular pattern formation remains largely unexplored, the intertwined pathways of auxin distribution, transport, and signal transduction have long been implicated in controlling all stages of both leaf and vein formation. Compared with Arabidopsis, Chinese cabbage has a unique morphological characteristic in its curling leaves in terms of the big proportion of leaf veins (Supplementary Figure S1). Moreover, a distinctly progressive curling was found for the Chinese cabbage leaf during leafy head formation, which was further confirmed by the finding that the cells in the curling veins showed an asymmetrical growth pattern (Figures 2K–P).

Leaf structure forms through development in three axes, and auxin is deeply involved in all of them; these axes are the proximal-distal (tip to base), the adaxial/abaxial (top to bottom) and the medial-lateral (middle to margin) (Bowman et al., 2002). The effects of auxin on leaf vein differentiation were mainly demonstrated in previous studies through its polar distribution in the proximal-distal axis (Sawchuk and Scarpella, 2013), while the developmental outcome of asymmetrical auxin distribution in the Ad/Ab axis is less reported. Compared with model plants, the prominent curling feature of Chinese cabbage leaves during heading provides us with an excellent model system in which to study the role of Ad/Ab auxin distribution in the leaf vein. We have shown that the growth of epidermal cells clearly shows Ad/Ab patterning, which is probably a consequence of the asymmetrical distribution of auxin in the leaf veins (Figures 2, 3). The supposition was strongly supported by the fact that the interruption of auxin transport by NPA leads to asymmetrical growth of abaxial/adaxial epidermal cells of leaf veins and consequent leaf curling at seedling stage (Figure 4). This is further reinforced by the fact that the genes that were differentially expressed between the adaxial vs. abaxial epidermal tissues of the curling midrib during heading were significantly enriched in the GO term “response to auxin” (Figure 5A). On the other hand, the results also indicated that the asymmetrical biosynthesis or transporting from the abaxial side to the adaxial side is important for the auxin asymmetrical distribution. Actually, the indication was supported by the fact that the IAA content in the abaxial cells was significantly higher than that of adaxial cells at the seedling stage (Figures 3I,J). Moreover, the supposition was also evidenced by Qi et al. (2014) in which the authors showed that transient adaxial low auxin domain is important for leaf polarity patterning. To test the hypothesis, they reasoned that if the adaxial side reversal of the polarity of auxin efflux transporter, PIN1, may lead to the adaxial low auxin domain. They then monitored DII-Venus signals (another auxin response marker) before and after auxin polar transport inhibitor 1-N-naphthylphthalamic acid (NPA) treatment. They then found that the boundary and adaxially enriched DII-Venus signal was evident in the first pair of true leaves 60 h after germination in Arabidopsis, however, the enriched DII-Venus signal mostly disappeared after a 5-h NPA treatment. Thus, auxin efflux is required for the transient adaxial low auxin domain formation.

According to BRAD,^1^ there are a total of 14 *PINs* and 142 *SAURs* in Chinese cabbage. And based on our RNA-seq data of Ad/Ab tissue of curling veins, the expression of 9 *PINs* and 117 *SAURs* could be detected (Supplementary Table S6, genes undetectable are marked with grey background color). Among them, only *PIN5*, *PIN7*, *SAUR19-24*, *SAUR32*, *SAUR50*, *SAUR62*, and *SAUR64-66* expressed adaxially/abaxially in leaf veins (Supplementary Table S6, marked with red color). The reason for why we chose *BrSAUR21* and *BrPIN5* as the candidate heading genes is that they are the only two genes of the above that are also in the region of BSA and QTL mapping interval (Figures 5D–F). *SAUR*s are a class of auxin early response genes with more than 70 members in Arabidopsis (Hagen and Guilfoyle, 2002). AtSAUR21 belongs to the SAUR19–24 subfamily, which are positive regulatory factors for cell extension that are induced by auxin (Spartz et al., 2012). Previous studies in Chinese cabbage have shown that multiple *BrSAURs* are involved in regulating the formation of leafy heads and head types (Gu et al., 2017; Li et al., 2019a,b), but the regulation mechanism remains unclear. Most *SAUR* genes contain auxin response elements in their promoter regions (Hagen and Guilfoyle, 2002). In a natural population of *B. rapa* with at least four morphotypes, the dominant haplotype of *BrSAUR21* is significantly enriched in Chinese cabbage (Figure 7), suggesting that this *BrSAUR21* haplotype was selected during Chinese cabbage domestication. Moreover, we found that most sequence variations present in the promoter region of *BrSAUR21* indicate that the *BrSAUR21* promoter might have contributed more to leafy head formation (Figure 7A; Supplementary Figure S9A). PINs are polar auxin transporter proteins. In Arabidopsis, there are eight PINs: PIN1-PIN8. Unlike the typical PINs, PIN5 is not directly involved in auxin cell-to-cell transport but regulates intracellular auxin homeostasis and metabolism (Mravec et al., 2009). PIN5, which antagonizes the functions of PIN6 and PIN8, regulates vascular development (Sawchuk et al., 2013). We found that AtPIN5 universally expressed in leaf veins by visualizing *AtPIN5::GUS* transgenic plants (Figures 7E–I). Together with the findings that *PIN5*-overexpression lines showed distinctive curling leaves and that *BrPIN* genes, such as *BrPIN1*, *BrPIN4*, and *BrPIN6*, were reported to be involved in the formation of leafy head (Mravec et al., 2009; Gao et al., 2017; Li et al., 2019a,b), we propose that *PIN5* functions in leaf vein development and consequently in leafy head formation. This is supported by the recent study of Cai et al. (2021), who found that another PIN protein, BrPIN3.3, is significantly associated with the leafy head in domesticated Chinese cabbage. They showed that for *BrPIN3.3*, a 279-bp deletion was found in 300 of 329 Chinese cabbage accessions, while it was present in only two non-heading accessions. This data suggests that the Ad/Ab patterning of auxin distribution and auxin gene expression in leaf veins functions in leafy head formation in Chinese cabbage. However, as we all know, PIN5 is a noncanonical PIN and resides in the endoplasmic reticulum, not the plasma membrane. As such it thus cannot contribute directly to auxin gradients or polar auxin flow in leaf vein. We proposed it might become elevated in areas of high IAA concentration to assist auxin homeostasis and finally help asymmetric growth of the Ad/Ab cells in leaf veins. We hope to uncover the underlying mechanism of leaf vein curling through further functional verification of *BrSAUR21* and *BrPIN5* in future research. Besides, as *B. rapa* is an important vegetable and oil crop worldwide and auxin is the fundamental phytohormone in plant growth, the DR5::GUS line of Chinese cabbage will provide the *B. rapa* community a useful tool to deepen mechanical investigations of plant morphogenesis, stress response, and organ development.

## Materials and Methods

### Plant Materials

A collection of 460 *B. rapa* inbred lines, including 210 Chinese cabbage (ssp. *pekinensis*), 250 pak choi (ssp. *chinensis*, ssp. *chinensis* var. *wu-tsai* Lin., ssp. *chinensis* var. *narinosa* Lin., ssp. *chinensis* var. *parachinensis* Lin.), 66 turnip (ssp. *rapifera*), and nine ssp. *oleifera*, was used for haplotype analysis of *BrPIN5* and *BrSAUR21*. An F_2_ population generated from F_1_ plants of Chinese cabbage × turnip, consisting of 1,565 individuals, was used to identify genetic loci associated with heading by BSA analysis. Two segregating populations, DH80 and DH85, generated from F_1_ plants of “Chinese cabbage × turnip” and “Chinese cabbage × pak choi,” respectively, were used for haplotype analysis of *BrPIN5* and *BrSAUR21*.

Transgenic plants of Chinese cabbage and *Arabidopsis thaliana* expressing *DR5::GUS* and *AtPIN5::GUS*, respectively, were used for auxin and auxin gene patterning analysis. *DR5::GUS* transgenic plants were obtained by introducing the *DR5::GUS* vector into a Chinese cabbage inbred line *via Agrobacterium* strain GV3101 using the cotyledon petiole dipping method (Su et al., 2021). The *AtPIN5::GUS* transgenic Arabidopsis seeds were obtained from Le et al. (2014).

### Growth Conditions and Phenotyping

The *DR5::GUS* transgenic plants used for GUS staining, the F_2_ population used for QTL mapping, the two DH populations used for haplotype analysis of *BrPIN5* and *BrSAUR21*, and the six Chinese cabbage and three pak choi lines used for time-course transcriptome analysis were grown on a farm at Tongzhou, Beijing, in the autumns of 2019 and 2020. For leaf vein proportion analysis, the 2nd-3rd outer leaves at the rosette, folding, and heading stages were used for analysis (Supplementary Figure S1). Leaf vein proportion = leaf vein/whole leaves (fresh weight).

For GUS staining of *DR5::GUS* transgenic plants, plants were grown on a farm under natural conditions at Tongzhou, Beijing, in the autumn (from 1st, Sept. to 30th, Oct.) of 2020. Different stages of plants, including seedling (28 days old), rosette (44 and 50 days old, respectively), folding (57 days old) and heading (67 and 87 days old, respectively) stages, were used for assessment. The 1st-2nd innermost leaves and the 3rd-5th outermost leaves of the above plant were harvested for GUS staining and photography. Transgenic *PIN5::GUS* Arabidopsis seedlings were planted in a growth chamber on a 16/8-h light/dark cycle (light intensity = 120 μmol·m^−2^ s^−1^) at 22°C; 19-day-old seedlings were used for GUS staining.

### NPA-Feeding

The Chinese cabbage seedlings were grown in 9 × 9 × 9 cm pots in the autumn of 2021 under natural conditions. 40-day-old seedlings were used for NPA-feeding and phenotype observation. There are nine plants for control group and nine plants for treatment group, and three biological replicates were performed. NPA solution (100 μmol/l + 1/10000 Tween20) and mock (as a control, water +1/10000 Tween20) were sprayed on the whole plant. During the experiment, the NPA and mock treatments were sprayed twice at an one-day interval. After 7 days of treatment, the plants were used for photography and phenotypic analysis.

### RNA Extraction, RNA-Seq, and qRT-PCR

Total RNA was extracted from the test materials using the RNAprep Pure Plant Pius Kit (Tiangen Biotech). The RNA was incubated with DNase I (Takara) and quantified using a NanoDrop 2000 (Thermo Fisher Scientific). A PrimeScript First Strand cDNA Synthesis Kit (Takara) was used for reverse transcription reaction.

Real-time PCR was executed on a LightCycler^®^ 480 real-time PCR machine (Roche Diagnostics, China). The Chinese cabbage GADPH gene was used as an internal standard to normalize gene expression (Su et al., 2018). The results were analyzed using the −2^ΔΔCt^ method (Livak and Schmittgen, 2001). For all experiments, three biological replicates were performed and within each biological replicate at least three technical replicates were used. Each qPCR sample (Figures 3K,L, 6; Supplementary Figures S4, S6, S7) shown in Supplementary Figure S3F (Ad/Ab) was mix tissue of at least 20 different leaf veins. All qPCR The RT-PCR primers are listed in Supplementary Table S1.

For time-course transcriptome analysis, the tips of the 2nd-3rd leaves of 21-, 29-, 38-, and 65-day-old plants of Chinese cabbage (see details in Supplementary Figure S5E) were harvested and stored at −80°C until use. For DEG analysis of Ab/Ad surface cells, the outmost surface tissues (shown in Supplementary Figure S3) of both sides of before-curling (~40-day-old) and curling (~65-day-old) leaves were dissected from the leaf veins as thin as possible and collected and stored at −80°C until use. In each sample, at least 20 dissected outmost tissues from different leaf veins were mix and used for analysis. RNA-seq was performed by Tiangen, and gene expression level analysis was performed with HTSeq software *via* union mode.

### BSA and QTL Mapping

For bulked segregant analysis, an F_2_ population consisting of 1,565 individuals derived from F_1_ plants of “Chinese cabbage × turnip” was used to screen loci that are genetically linked with heading. Individuals with heading and non-heading phenotypes, respectively, from the F_2_ population were selected and used for constructing the two DNA bulks for BSA sequencing. DNA bulks of the heading- and non-heading-progeny plants consisting of 111 and 81 individuals, respectively, were used for sequencing. Sequencing, SNP-calling, and calculation of the SNP-index and *Δ*(SNP-index) were performed as described previously (Su et al., 2021).

The DH80 population (Su et al., 2018) was used for QTL mapping which was implemented using the composite interval mapping function embedded in MapQTL 5.0 (Van Ooijen, 2004). QTL peaks with LODS (Log of Odds)≧3.0 were supposed to be significantly associated with heading. A 0.05 significance level and 1,000 permutations applied during QTL mapping.

### GUS Staining

For GUS staining, leaves from 28-, 44-, 50-, 57-, 67-, and 87-day-old T_2_ generation *DR5::GUS* transgenic Chinese cabbage seedlings were collected in 90% acetone on ice. The 1st-2nd innermost and the 3rd-5th outermost leaves of at least 5 different plants of each stage were used. GUS assays were performed as previously described with modifications (Su et al., 2018; Yue et al., 2019).

The Chinese cabbage leaves were incubated in valve bags (31 × 21 cm) in 90% acetone for 30 min at 4°C. The samples were then bathed with phosphate buffer (*p*H7.2) for three times for 5 min per session. Phosphate buffer was then substituted with GUS assay buffer (0.5 mM K_4_[Fe(CN)_6_].3H_2_O, 0.5 mM K_3_[Fe(CN)_6_], and 0.1% Triton X-100 in phosphate buffer), and the samples were put under vacuum for 15 min. The GUS assay buffer was then replaced by a GUS reaction buffer (with 1.9 mM X-Gluc added in the assay buffer) and vacuumized for 30 min. Samples were then incubated overnight at 37°C in dark, after which the reaction was blocked by replacing the reaction buffer with a fixative solution (4% glutareldehyde and 10.8% formaldehyde). After fixation for overnight at 4°C, the samples were cleared with 95% ethanol and transferred to absolute lactic acid. For microscopy, plantlets were mounted on an object slide and covered with a drop of lactic acid and a coverslip.

### Measurement of Endogenous IAA by LC–MS/MS

Ad/Ab epidermal cells used for the RNA-seq above were used for IAA content measurement. The samples (0.5 g) were homogenized in liquid nitrogen and incubated in a precooled extraction buffer (isopropanol: water: hydrochloric acid = 2:1:0.002, volume ratio). The mixtures were kept at 4°C for 16 h, shaken twice, and centrifuged at 15,000 r/min for 5 min at 4°C. The supernatant was collected and re-extracted as the above. The supernatant was then freeze-dried and dissolved in 50% methanol. LC–MS/MS was performed by using a SHIMADZU-20A HPLC system (Shimadzu Corp., Kyoto, Japan) coupled with a 6,500 triple quadrupole mass spectrometer to detect IAA contents.

### Scanning Electron Microscopy, Semi-thin Sectioning, and Freehand Sectioning

Morphological observation of Ad/Ab epidermal cells of leaf veins was conducted using an ultra-high-resolution scanning electron microscope (Cryo-SEM Regulus8100; Hitachi). The Ad/Ab cells in the same location were chosen for observation. For semi-thin sectioning, GUS-stained tissue samples were transversely sectioned into 5 μm slices and then stained with PAS and examined under a light microscope. However, due to the limited thickness, we were unable to photograph the GUS signal in the transverse vein sections. Alternatively, samples were manually sectioned into 5 mm slices and observed under a stereoscopic light microscope.

### Cell Number Ratio and Size Calculate

In Figure 2Q and Supplementary Figure S2: for cell number counting, Ad/Ab epidermal cells in the same location shown in the red box of Figures 2B,J were chosen for observation and cell number counting, respectively. In before-curling leaves, an area of 0.125 mm^2^ of the primary leaf vein and areas of 0.617 mm^2^ of the midrib areas, midrib1 and midrib2 for each, were used for cell number assessment of Ad/Ab epidermal cells; six different areas of primary veins, seven different areas of midrib1, and 23 different areas of midrib2 were used for cell number counting and statistical analyses. The corresponding areas used in curling leaves were both 1.187 mm^2^; eight different areas of primary veins, eight different areas of midrib1, and 12 different areas of midrib2 were used for cell number counting and statistical analyses.

In Figure 4 and Supplementary Figure S8: Ad/Ab epidermal cells in the same location shown in the red box of Figures 4E,F and Supplementary Figure S8C were chosen for observation and cell number counting. For Chinese cabbage (Figures 4K–M), cell numbers in the area of 0.134 mm^2^ (Mock, Figure 4K) and 0.093 mm^2^(treated with 100 μM NPA, Figure 4M) were counted; a total of 32 and 48 areas, respectively, were used for counting and statistical analyses. For Pak choi (Supplementary Figures S8F,G), a total of 48 areas (each are = 0.176 mm^2^) were used for cell number assessment.

Cell number ratio was calculated by Ab/Ad cell numbers.

In Figure 4; Supplementary Figures S2, S8: cell size was calculated as the area/cell number of the counting zone.

### Statistical Analyses

SPSS was used for statistical analysis. Statistical significance was calculated using one-way ANOVA or Student’s *t*-test. Different alphabets represent the significance difference at the *p* ≤ 0.01 level; asterisks indicate significant differences using Student’s *t*-test ^**^p < 0.01. The sample size was evaluated by SPSS and shown in each figure or supplemental files.

## Data Availability Statement

The original contributions presented in the study are included in the article/Supplementary Material, further inquiries can be directed to the corresponding authors.

## Author Contributions

XY and TS designed and carried out the experiments. XX, PL, WW, YY, DZ, XZ, JW, LS, and GJ contributed experimental materials. TS, SY, and FZ wrote the paper. All authors contributed to the article and approved the submitted version.

## Funding

This work was supported by grants from the Natural Science Foundation of China (32172557), the Scientist Training Program of BAAFS (JKZX202206), the Innovation and Capacity-Building Project of BAAFS (KJCX20200204 and KYCX20210427), and the Collaborative Innovation Center of BAAFS (KJCX201907-2).

## Conflict of Interest

The authors declare that the research was conducted in the absence of any commercial or financial relationships that could be construed as a potential conflict of interest.

## Publisher’s Note

All claims expressed in this article are solely those of the authors and do not necessarily represent those of their affiliated organizations, or those of the publisher, the editors and the reviewers. Any product that may be evaluated in this article, or claim that may be made by its manufacturer, is not guaranteed or endorsed by the publisher.

## Footnotes

^1^ http://brassicadb.cn/
