# Supplementary material for: The Adaxial/Abaxial Patterning of Auxin and Auxin Gene in Leaf Veins Functions in Leafy Head Formation of Chinese Cabbage
Source: Front Plant Sci. 2022 Jun 9;13:918112. doi: 10.3389/fpls.2022.918112 (PMC9224592; doi:10.3389/fpls.2022.918112)
Supplement: Supplementary file 1 [file Data_Sheet_1.docx]

**Supplementary data:**

**
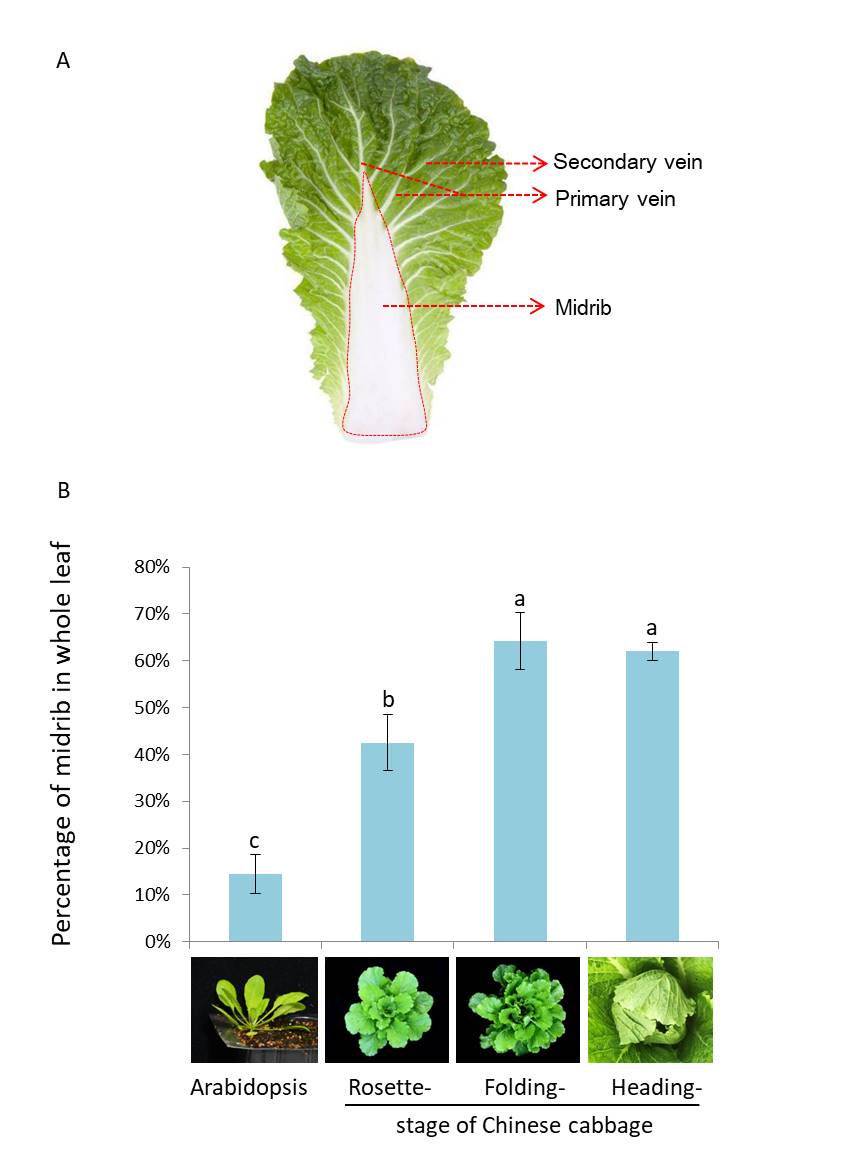
**

**Fig. S1 The midrib percentage in the whole leaf.**

(**A**) Vein system of the *B. rapa* leaf. (**B**) The innermost 2^nd^-3^rd^ leaves and the corresponding midribs of each plant were weighed: Arabidopsis (~25-day old), pak choi (~30-day old), rosette- (~48-day), and heading- (80-day old) stages of Chinese cabbage. Statistical analysis was performed using one-way ANOVA followed by the LSD test. Values are means ± SD (n = 14-21).


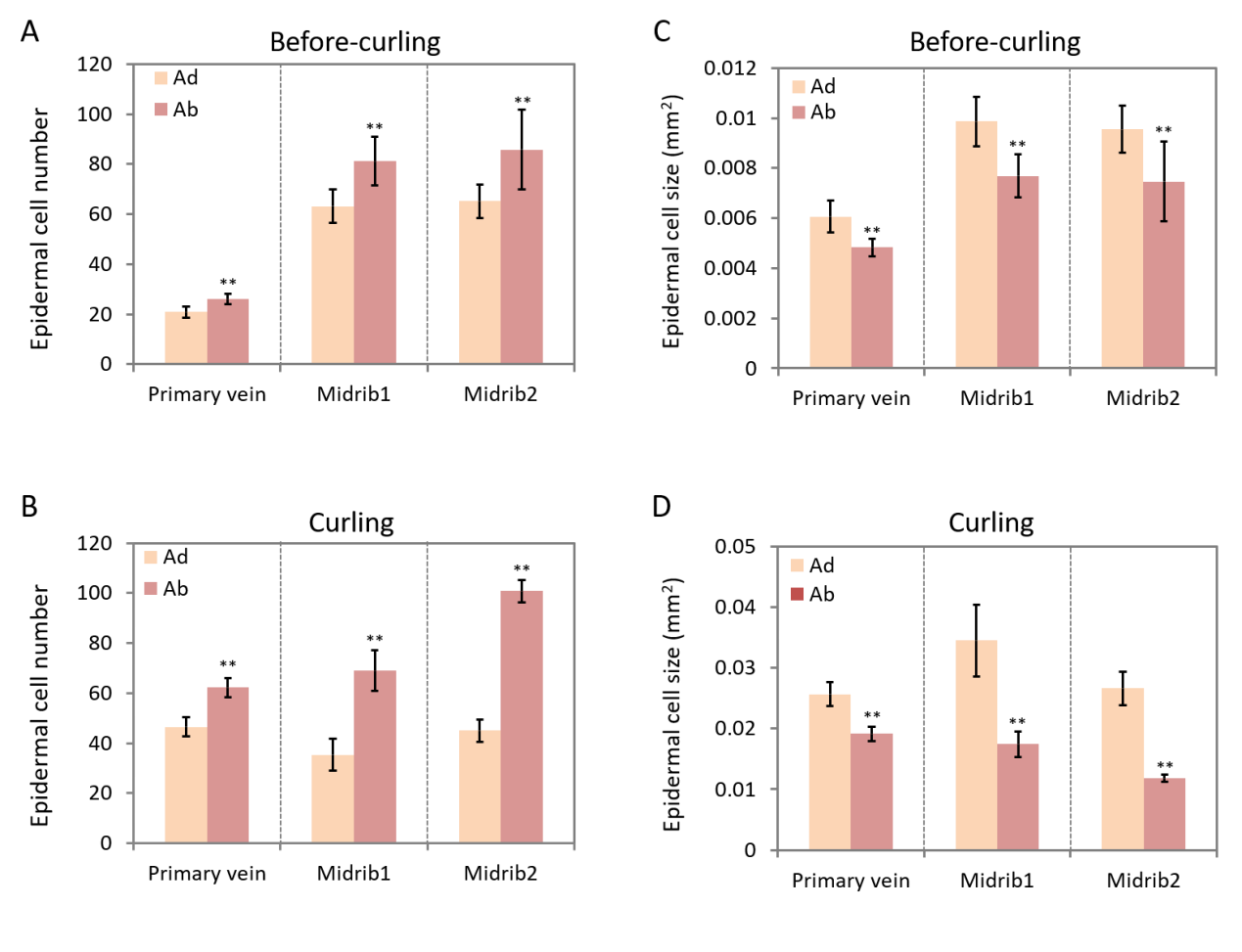


**Fig. S2 The abaxial epidermic cell number is more than that of the adaxial side in Chinese cabbage leaf veins.**

**A** and **B,** Abaxial and adaxial epidermic cell numbers of the primary vein, higher midrib part, and lower midrib part, respectively. Cell numbers were counted as described in “Materials and methods-- Cell number ratio and size calculate”. **C** and **D,** the cell size assessment of the corresponding areas shown in A and B. Values are means ± SD (n = 6-23). Asterisks indicate signiﬁcant differences using Student’s t-test ***p* < 0.01. Ad/Ab, adaxial/abaxial.


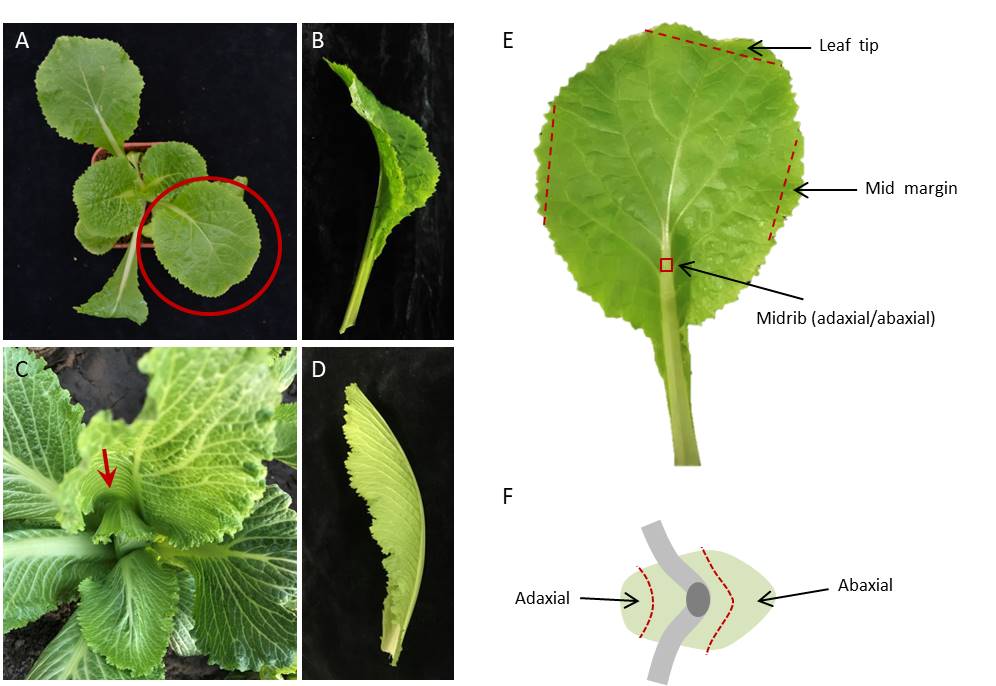


**Fig. S3 The leaf materials used in qPCR and RNA-seq.**

**A** and **B** are the before-curling (~40-day old) plants, and **C** and **D** are curling (~65-day old) plants and leaves used in qRT-PCR and RNA-seq. **E** shows the parts of the leaf, the tip and mid-margin (dashed red line), that were used in qRT-PCR and RNA-seq; the midribs were used in transverse sectioning, qRT-PCR, and RNA-seq. **F** showed the vein surface tissues of both abaxial and adaxial sides which were dissected and used in qRT-PCR and RNA-seq. The marked tissues in E and F were materials used in Fig. 3I- L, Fig.6 and Fig. S7.


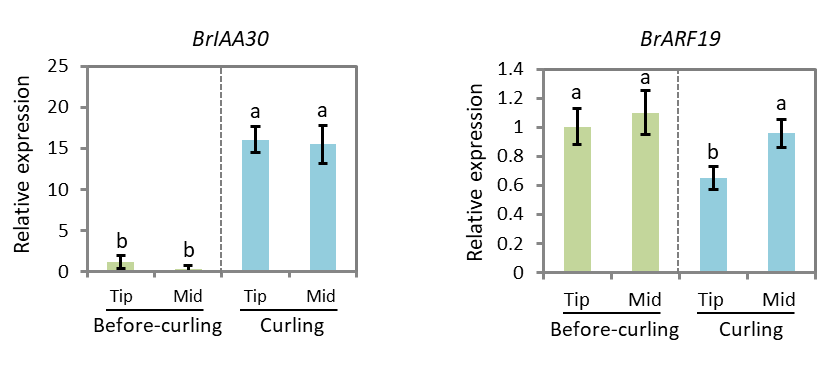


**Fig. S4 Relative expression of *BrIAA30* and *BrARF19* in leaf margins of before-curling and curling plants.**

Error bars represent the standard errors derived from three replicates. Different letters represent highly significant differences as determined by Student’ s t-test, P≤0.01. The materials used were from the plant parts shown in Fig. S3E and F.


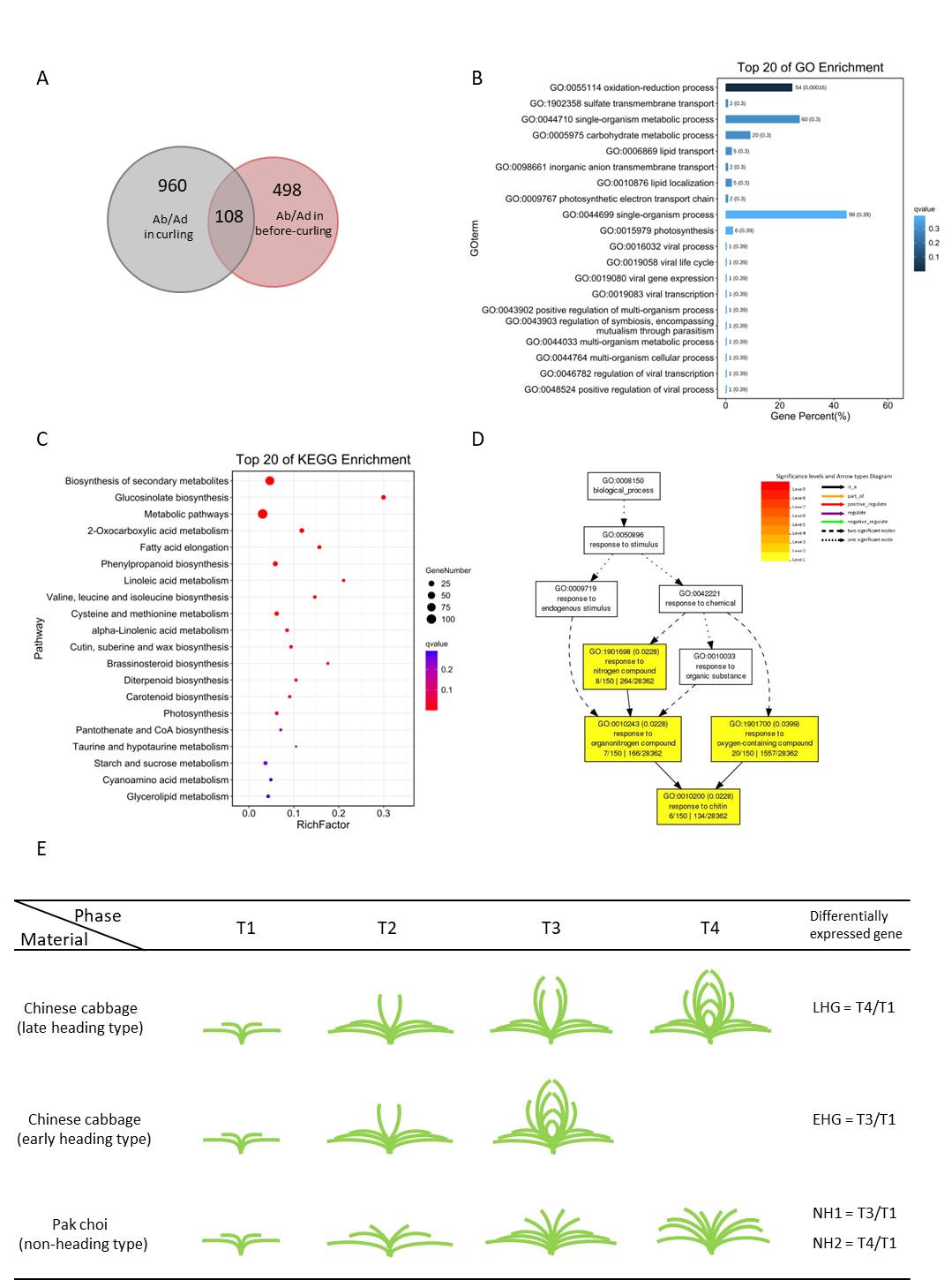


**Fig. S5 Identification of auxin genes specially expressed during leafy head formation using RNA-seq.**

**A**: the venn diagrams of Ab/Ad DEG numbers of curling and before-curling leaf veins based on RNA-seq data (for materials see Fig. S3D, F). GO (**B**) and KEGG pathway (**C**) analyses of the Ab/Ad DEGs of before-curling leaf veins (for materials see Fig. S3B, F). **D**: GO analysis of DEGs of different comparisons of different *B. rapa* crops during the farming season (see materials and analysis flow chart in Fig. S3E and Fig. S5E) by agriGO. **E**: the sampling and analysis method used for RNA-seq, T1-T4 represent the plant developmental phases at 21, 29, 38, and 65 days, respectively. EHG, the differentially expressed genes in the early heading-type materials (*B. rapa*); LHG, the differentially expressed genes in the late heading-type materials (*B. rapa*); NH, the differentially expressed genes in the non-heading materials (pak choi). DEG, differentially expressed gene; GO, Gene Ontology; KEGG, Kyoto Encyclopedia of Genes and Genomes.


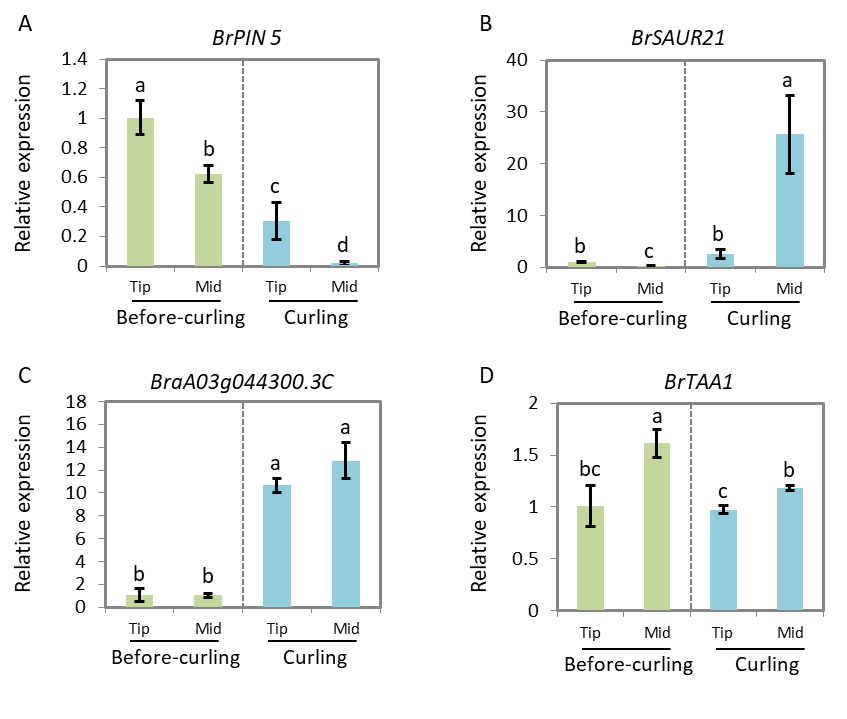


**Fig. S6 Expression of *BrPIN5* and *BrSAUR21*, as well as *BraA03g044300.3C*and *BrTAA1* in leaf tips and middle margins in before-curling and curling leaves.**

For materials see Fig. S3E. Error bars represent the standard errors from three replicates. Different letters represent highly significant differences as determined by Student’ s t-test, *p*≤0.01.

**
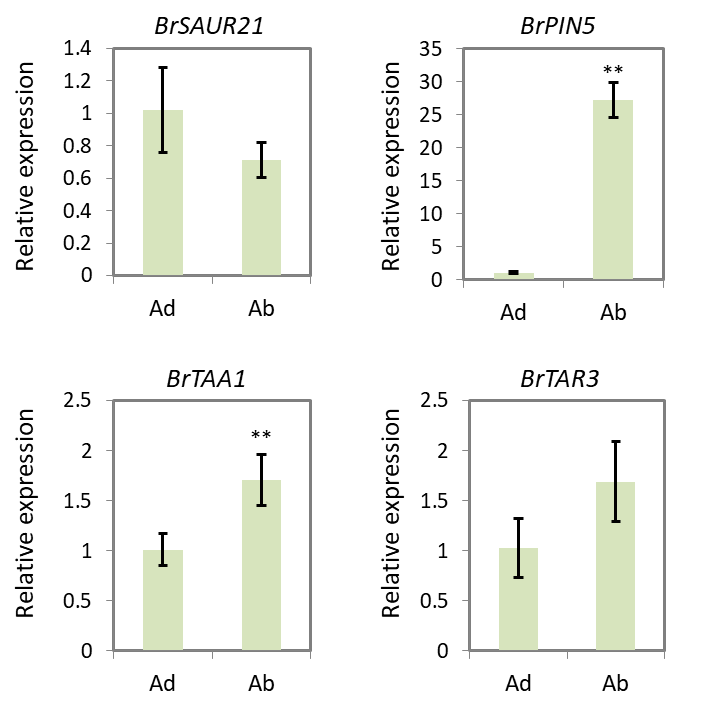
**

**Fig. S7 Expressions of *BrPIN5* , *BrSAUR21, BrTAA1* and *BrTAR3* in** **adaxial and abaxial epidermal cells of leaf veins in pak choi.**

Error bars represent the standard errors derived from three replicates. Asterisks indicate signiﬁcant differences using Student’s t-test ***p*≤0.01 Ad/Ab, adaxial/abaxial.

**
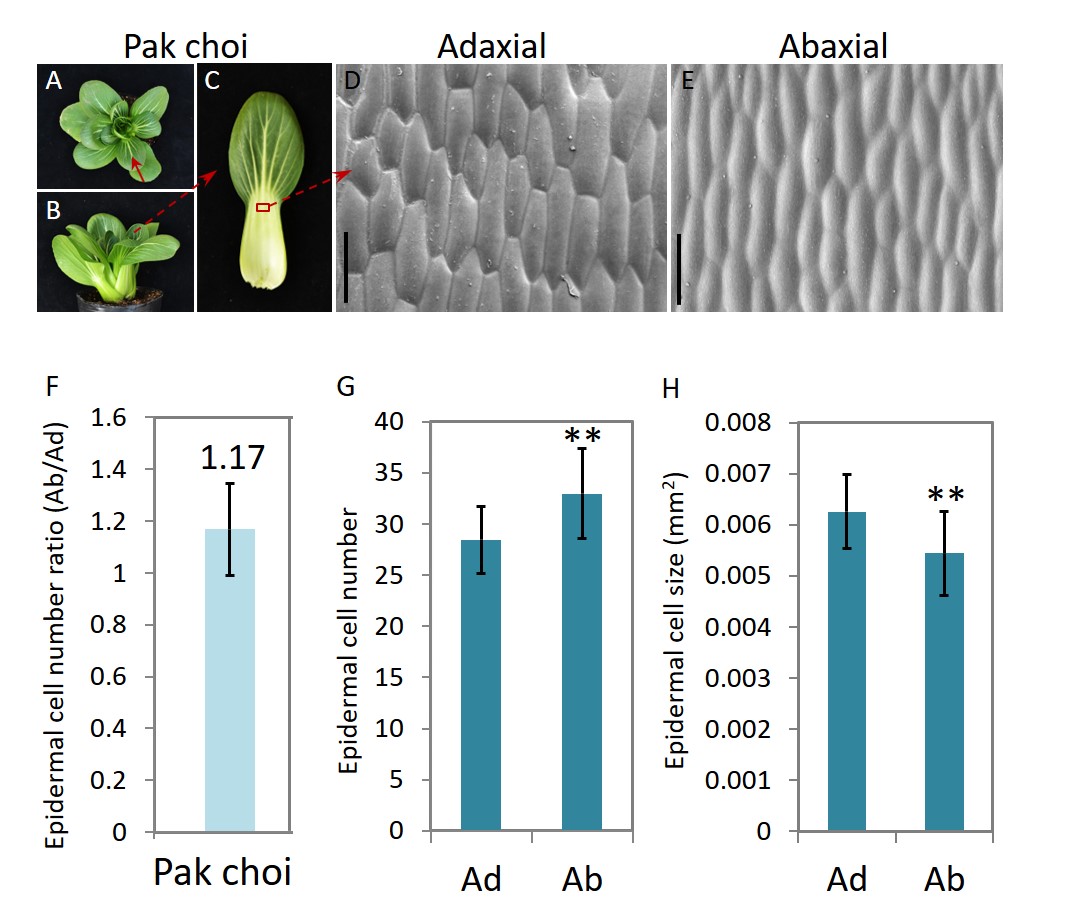
**

**Fig. S8 Cells in veins of pak choi show a mild asymmetrical growth pattern**

Morphological observation of the Ad/Ab epidermic cells of the leaf veins in pak choi (~28-day-old); **A**-**C**, pictures of the observed plant and leave; **D** and **E,** scanning electron micrographs of the Ad/Ab cells of the midrib part marked with a red squarer. **F**, the cell number ratios (Ad/Ab) of leaf vein epidermis. **G,** the abaxial and adaxial epidermic cell numbers of the midrib area displayed in C. The epidermis area used for calculation was 0.176 mm^2^. **H**, the cell size of above corresponding areas. Ab/Ad, abaxial/adaxial; Values are means ± SD (n = 48). Asterisks indicate signiﬁcant differences using Student’s t-test ***p* ≤0.01. Scale bars: 100 μm ( **D-E**).

**Fig. S9 Haplotype analysis of *BrSAUR21* and *BrPIN5* in natural lines of Chinese cabbage.**

Pos. denotes the position of an SNP or InDel from the start codons (+1) of *BrSAUR21* and *BrPIN5*. Hap denotes the haplotypes of *BrSAUR21* and *BrPIN5*. n denotes the number of lines belonging to each haplotype group.


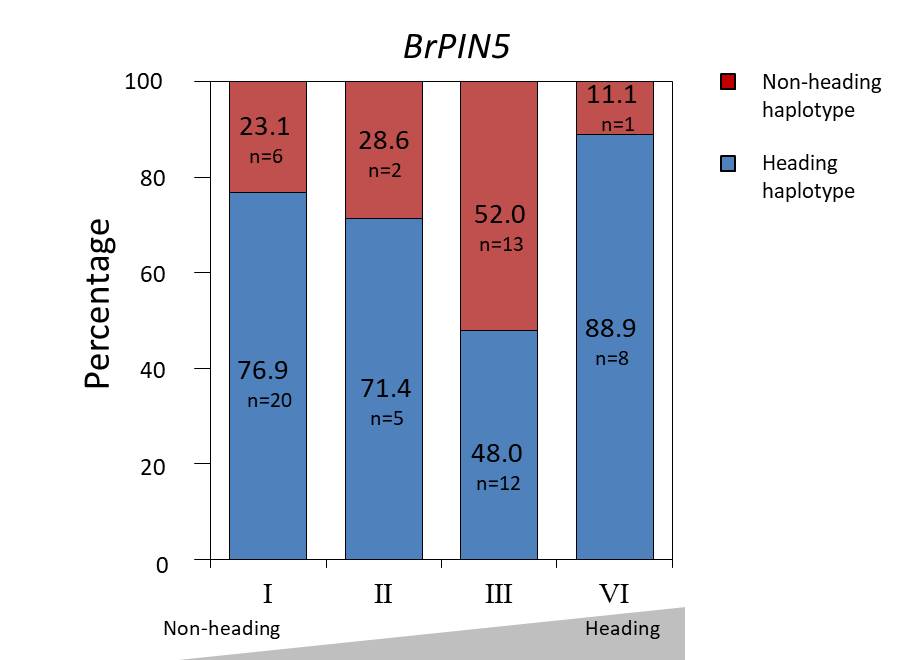


**Fig. S10 Haplotype analysis of *BrPIN5* in the DH80 segregation population**

Haplotype analysis of *BrPIN5* was performed based on the SNPs and Indels in the gene bodies. The heading haplotype of *BrPIN5* was defined as the haplotype that is from Chinese cabbage; I to IV indicate the heading levels from non-heading to heading.

**Table S1 Primers used for qRT-PCR**

| **Gene** | **Primer** | **Sequence (5'-3')** |
| --- | --- | --- |
| BraA07g024980.3C | BrIAA30-qF | CTCGGACTCAGCTTCGGCTC |
|  | BrIAA30-qR | CATGATCATTTCTTGATCAGCAGCC |
| BraA08g028300.3C | BrARF19-qF | ACAAGATCAAGTTAGAATGACGAATC |
|  | BrARF19-qR | GCTTGGAAAGTACATTATGAAGACAC |
| BraA10g023220.3C | BrPIN5-qF | GCATATCTTCACTCGTGATC |
|  | BrPIN5-qR | CTAGTAATGGACCAGCAATAG |
| BraA10g022010.3C | BrSAUR21-qF | ACTGGAGCCTAGACGTTAAG |
|  | BrSAUR21-qR | GCTTCTTCCACGAGCAAAAC |
| BraA03g044300.3C | Br012970-qF | GGTCATGAGGAGCTTTGGTTG |
|  | Br012970-qR | GGTAAGAGTAATGGCCAGTG |
| BraA02g019880.3C | BrTAA1-qF | GTCTGACTCCATCATCAACC |
|  | BrTAA1-qR | GTAACGATCCTCGGTTGCTG |
| BraA09g031290.3C | BrTAR3-q-F1 | CTACAATGTGTACAGAGAAC |
|  | BrTAR3-q-R1 | GGTGTGAAATGAGGCCAGTAG |

**Table S2 Auxin-related DEGs obtained from RNA-seq data of Ab/Ad tissues of curling leaf veins.**

| **Gene in Arabidopsis** | **Gene_id** | **log2FoldChange** | ***p*val** |
| --- | --- | --- | --- |
| *SAUR19* | BraA03g008240.3C.1 | -2.357780297 | 0.0049 |
|  | BraA10g022110.3C.1 | -1.924704387 | 0.033213 |
| *SAUR20* | BraA10g022050.3C.1 | -2.917442483 | 0.035429 |
|  | BraA10g022060.3C.1 | -3.665560938 | 0.00015 |
|  | BraA10g022080.3C.1 | -2.807817992 | 0.000837 |
|  | BraA10g022090.3C.1 | -3.896768915 | 0.005862 |
|  | BraA10g022020.3C.1 | -3.770561823 | 0.000275 |
|  | BraA03g008270.3C.1 | -2.5478795 | 0.004167 |
|  | BraA03g008290.3C.1 | -2.947907764 | 0.001825 |
|  | BraA10g022120.3C.1 | -3.074463652 | 0.000791 |
|  | BraA02g035550.3C.1 | -2.432833742 | 0.008553 |
| *SAUR21* | BraA03g008250.3C.1 | -3.262640358 | 0.007289 |
|  | BraA03g008260.3C.1 | -3.266482424 | 0.015853 |
|  | BraA03g008300.3C.1 | -Inf | 0.002748 |
|  | BraA03g008320.3C.1 | -2.020882611 | 0.037608 |
|  | BraA10g022010.3C.1 | -4.577626843 | 8.35E-06 |
|  | BraA10g022040.3C.1 | -3.764461623 | 0.030207 |
|  | BraA10g022100.3C.1 | -2.544783586 | 0.044246 |
|  | BraA02g007500.3C.1 | -3.484702982 | 5.96E-05 |
| *SAUR22* | BraA02g007410.3C.1 | -3.994518706 | 0.000558 |
| *SAUR23* | BraA02g007460.3C.1 | -3.10149661 | 0.00376 |
| *SAUR24* | BraA03g008280.3C.1 | -5.466781074 | 0.000881 |
| *SAUR32* | BraA09g000950.3C.1 | 1.432027175 | 0.044691 |
| *SAUR50* | BraA01g000150.3C.1 | -2.100242093 | 0.005503 |
|  | BraA01g003540.3C.1 | -2.891278903 | 0.008665 |
|  | BraA03g059020.3C.1 | -4.851444925 | 0.001917 |
|  | BraA06g044670.3C.1 | -3.739799569 | 0.001854 |
| *SAUR62* | BraA07g039670.3C.1 | 3.925536348 | 0.048461 |
| *SAUR64* | BraA08g023840.3C.1 | -2.771342037 | 0.002528 |
| *SAUR65* | BraA08g023860.3C.1 | -2.506309439 | 0.035147 |
| *SAUR66* | BraA08g023850.3C.1 | -3.172495735 | 0.006644 |
| *IAA2* | BraA05g024120.3C.1 | -1.547999425 | 0.022037 |
| *IAA3* | BraA10g002780.3C.1 | -3.125272611 | 2.41E-05 |
| *IAA5* | BraA09g058640.3C.1 | -Inf | 1.2E-05 |
| *IAA6* | BraA06g001750.3C.1 | -3.045048923 | 0.002515 |
| *IAA14* | BraA08g010620.3C.1 | -3.201919315 | 5.77E-05 |
| *IAA15* | BraA07g043040.3C.1 | -Inf | 0.014493 |
| *IAA17* | BraA08g034980.3C.1 | -2.004480675 | 0.003109 |
|  | BraA10g002790.3C.1 | -1.985088836 | 0.003421 |
| *IAA19* | BraA05g031540.3C.1 | -2.107293019 | 0.002486 |
|  | BraA03g036990.3C.1 | -2.392487292 | 0.000924 |
| *ABCB19* | BraA09g003610.3C.1 | -2.326182744 | 0.001509 |
| *PIN5* | BraA10g023220.3C.1 | 2.511959806 | 0.006056 |
| *PIN7* | BraA07g014360.3C.1 | -2.77752937 | 0.000419 |
| *PID* | BraA05g010440.3C.1 | -2.641867781 | 0.000862 |
| *D6PKL2* | BraA06g021340.3C.1 | -3.309099669 | 0.000279 |
| *TAR4* | BraA08g008240.3C.1 | 4.063039872 | 0.007419 |
| *IAR3* | BraA06g002370.3C.1 | -2.031922134 | 0.023747 |
|  | BraA04g009650.3C.1 | 1.404537537 | 0.035279 |
|  | BraA04g014870.3C.1 | 4.577613045 | 0.002327 |
|  | BraA06g004330.3C.1 | 5.247464443 | 0.036873 |

**Table S3 Auxin-related DEGs obtained from RNA-seq data using different leaf materials during heading formation.**

| **Gene in Arabidopsis** | **Gene_id** | **log2FoldChange**  **(EHG=T3/T1)** | **log2FoldChange (LHG=T4/T1)** |
| --- | --- | --- | --- |
| *REV1* | BraA03g007900.3C | 2.077534623 | 2.628861736 |
| *CIPK6* | BraA01g006800.3C | -1.066590487 | -1.029371489 |
| *PHOT1* | BraA06g023540.3C | 1.254106874 | 1.308445229 |
| *WAT1* | BraA07g039050.3C | 1.516266403 | 1.443858034 |
| *NF-YB2* | BraA09g022870.3C | -1.093231243 | -1.026318165 |
| *SAUR34* | BraA08g015020.3C | -3.181464694 | -5.017127125 |
| *SAUR21* | BraA10g022010.3C | -1.623910942 | -3.461105035 |
| *AT5G61290* | BraA03g044300.3C | -2.410630531 | -2.96138801 |

**Table S4 Chromosomal regions related with leafy head formation identified by BSA.**

| **Chr** | **Region** |
| --- | --- |
| A01 | 10890000-23070000 |
| A02 | 28440000-30170000 |
| A03 | 18090000-20210000 |
| A03 | 20480000-22960000 |
| A08 | 1030000-2350000 |
| A09 | 37900000-44070000 |
| A10 | 0-10310000 |
| A10 | 15440000-16460000 |

**Table S5 95% confidence interval genes obtained from bulked segregant analysis (BSA) on Chromosomes A03 and A10 .**

Please see the attached Table S5 file.

**Table S6 Expressions of *PIN* and *SAUR* genes from RNA-seq data of abaxial/adaxial tissue from before-curling and curling leaf veins.**

| Gene | Gene id (v1.5) | Gene id (v3.0) | FPKM | | | | |
| --- | --- | --- | --- | --- | --- | --- | --- |
|  |  |  | Before-curling | |  | Curling | |
|  |  |  | Ab | Ad |  | Ad | Ab |
| PINs | Bra016366 | BraA08g026700.3C.1 | 0 | 0 |  | 0 | 0 |
|  | Bra008615 | BraA10g023220.3C.1 | 29.93041 | 23.87462 |  | 1.967628 | 12.00819 |
|  | Bra023503 | BraA02g005840.3C.1 | 7.649912 | 8.060743 |  | 4.03115 | 2.318485 |
|  | Bra006834 | BraA03g012590.3C.1 | 0 | 0 |  | 0 | 0 |
|  | Bra003938 | BraA07g029730.3C.1 | 160.4437 | 126.9347 |  | 25.1443 | 13.33077 |
|  | Bra012358 | BraA07g014360.3C.1 | 21.89444 | 19.7438 |  | 15.79076 | 2.230778 |
|  | Bra008722 | BraA10g024250.3C.1 | 0.104857 | 0 |  | 0 | 0.059028 |
|  | Bra026669 | BraA01g029980.3C.1 | 0 | 0 |  | 0 | 0 |
|  | Bra015694 | BraA07g040560.3C.1 | 1.938118 | 1.738241 |  | 3.011425 | 1.940828 |
|  | Bra002763 | BraA10g014980.3C.1 | 0 | 0 |  | 0 | 0 |
|  | Bra035648 | BraA02g012510.3C.1 | 0 | 0 |  | 0 | 0 |
|  | Bra016173 | BraA07g035620.3C.1 | 97.35617 | 61.7878 |  | 11.79412 | 10.587 |
|  | Bra008105 | BraA02g021850.3C.1 | 11.24219 | 10.92487 |  | 2.781142 | 3.014522 |
|  | Bra015983 | BraA07g037630.3C.1 | 7.460291 | 10.15024 |  | 4.417887 | 2.897843 |
| SAURs | Bra004515 | BraA05g001150.3C.1 | 48.96924 | 43.21699 |  | 3.755878 | 11.98282 |
|  | Bra039345 | BraA01g038610.3C.1 | 2.646552 | 0.927548 |  | 0 | 0.404902 |
|  | Bra033580 | BraA06g044680.3C.1 | 0 | 0 |  | 0.334103 | 0.125224 |
|  | Bra002125 | BraA10g022070.3C.1 | 8.119713 | 11.81961 |  | 26.84242 | 7.155944 |
|  | Bra006984 | BraA09g044260.3C.1 | 0 | 0 |  | 0 | 0 |
|  | Bra023659 | BraA02g007460.3C.1 | 6.055652 | 5.724958 |  | 13.31791 | 1.414962 |
|  | Bra013061 | BraA03g043220.3C.1 | 0 | 0 |  | 0 | 0.185347 |
|  | Bra002122 | BraA10g022100.3C.1 | 4.739367 | 1.969156 |  | 14.42194 | 2.463638 |
|  | Bra023654 | BraA02g007410.3C.1 | 3.783849 | 4.533909 |  | 22.66562 | 1.310813 |
|  | Bra022763 | BraA03g015870.3C.1 | 0 | 0 |  | 0 | 0 |
|  | Bra034651 | BraA08g015730.3C.1 | 3.422876 | 4.233445 |  | 1.783466 | 0.126834 |
|  | Bra020127 | BraA02g009130.3C.1 | 0 | 0 |  | 0 | 0 |
|  | Bra011258 | BraA01g006580.3C.1 | 0 | 0 |  | 0 | 0 |
|  | Bra003746 | BraA07g027690.3C.1 | 0 | 0 |  | 0.501429 | 0 |
|  | Bra026598 | BraA02g035550.3C.1 | 6.082786 | 11.51274 |  | 22.01917 | 4.024949 |
|  | Bra005293 | BraA05g009190.3C.1 | 0 | 0.058779 |  | 0 | 0 |
|  | Bra029798 | BraA05g036760.3C.1 | 0.068856 | 1.074982 |  | 0.232775 | 3.09661 |
|  | Bra027984 | BraA09g019230.3C.1 | 0 | 0 |  | 0 | 0 |
|  | Bra015778 | BraA07g039670.3C.1 | 0.231189 | 0 |  | 0.347517 | 5.914263 |
|  | Bra003125 | BraA07g021150.3C.1 | 0 | 0 |  | 0 | 0 |
|  | Bra018403 | BraA07g015730.3C.1 | 0.570497 | 0.772305 |  | 1.365402 | 2.169909 |
|  | Bra023658 | BraA02g007450.3C.1 | 0.151159 | 0.18273 |  | 0.174867 | 0 |
|  | Bra007639 | BraA09g051400.3C.1 | 4.277772 | 4.562557 |  | 0 | 0.979531 |
|  | Bra029934 | BraA03g050310.3C.1 | 0 | 0.071218 |  | 0 | 0 |
|  | Bra017680 | BraA03g059070.3C.1 | 7.899207 | 5.060248 |  | 0.740239 | 0.705014 |
|  | Bra017211 | BraA04g026250.3C.1 | 0 | 0 |  | 1.449462 | 0 |
|  | Bra006433 | BraA03g008270.3C.1 | 8.870954 | 17.63796 |  | 46.56201 | 7.480659 |
|  | Bra002331 | BraA10g019850.3C.1 | 0 | 0 |  | 0 | 0.117778 |
|  | Bra015832 | BraA07g039120.3C.1 | 0.362817 | 0.844386 |  | 0.509037 | 0 |
|  | Bra034954 | BraA06g022270.3C.1 | 2.656637 | 3.461417 |  | 0.492373 | 0 |
|  | Bra032321 | BraA09g035410.3C.1 | 0 | 0 |  | 0.831477 | 0.084403 |
|  | Bra013630 | BraA01g013230.3C.1 | 0.312309 | 0.35464 |  | 25.06462 | 8.110311 |
|  | Bra006430 | BraA03g008240.3C.1 | 3.30848 | 2.633878 |  | 55.05903 | 10.1911 |
|  | Bra034654 | BraA10g022040.3C.1 | 9.194864 | 13.078 |  | 7.697402 | 0.491246 |
|  | Bra017677 | BraA08g015710.3C.1 | 3.393725 | 2.001475 |  | 0 | 0 |
|  | Bra011562 | BraA01g003520.3C.1 | 0.308785 | 0.200795 |  | 0.171399 | 0 |
|  | Bra006434 | BraA03g008280.3C.1 | 2.194096 | 1.836283 |  | 13.79198 | 0.361328 |
|  | Bra011563 | BraA01g003510.3C.1 | 5.284012 | 6.416757 |  | 0.299928 | 0.314332 |
|  | Bra032736 | BraA04g008290.3C.1 | 0.656815 | 0.177658 |  | 4.089758 | 2.158527 |
|  | Bra002121 | BraA10g022110.3C.1 | 16.1588 | 14.07062 |  | 39.36256 | 8.947822 |
|  | Bra026596 | BraA02g035570.3C.1 | 0.26777 | 0.58114 |  | 1.172872 | 0.796867 |
|  | Bra037178 | BraA09g009680.3C.1 | 2.750944 | 1.187454 |  | 0 | 0 |
|  | Bra019369 | BraA03g050310.3C.1 | 0 | 0.071218 |  | 0 | 0 |
|  | Bra006431 | BraA03g008250.3C.1 | 1.933163 | 5.332345 |  | 23.60097 | 1.921201 |
|  | Bra017254 | BraA04g025770.3C.1 | 0 | 0.596372 |  | 0 | 0 |
|  | Bra002126 | BraA10g022060.3C.1 | 12.35857 | 13.91747 |  | 35.39149 | 2.188803 |
|  | Bra025821 | BraA06g015990.3C.1 | 0 | 0 |  | 0 | 0 |
|  | Bra012231 | BraA07g015730.3C.1 | 0.570497 | 0.772305 |  | 1.365402 | 2.169909 |
|  | Bra035803 | BraA05g027060.3C.1 | 0.114844 | 0 |  | 0 | 0 |
|  | Bra011564 | BraA01g003500.3C.1 | 1.172852 | 0.405414 |  | 0 | 0.274035 |
|  | Bra034725 | BraA05g034250.3C.1 | 2.954866 | 0.992417 |  | 0.500503 | 1.024755 |
|  | Bra028556 | BraA02g003720.3C.1 | 0 | 0 |  | 0.076926 | 0.125461 |
|  | Bra023653 | BraA02g007400.3C.1 | 7.591957 | 10.0656 |  | 4.207882 | 0.621907 |
|  | Bra033579 | BraA06g044690.3C.1 | 13.97077 | 19.01782 |  | 18.09897 | 7.9319 |
|  | Bra000629 | BraA03g026350.3C.1 | 0 | 0 |  | 0 | 0 |
|  | Bra011979 | BraA07g018500.3C.1 | 8.821076 | 0.172958 |  | 0.639595 | 1.470389 |
|  | Bra010501 | BraA08g020500.3C.1 | 1.389368 | 6.665817 |  | 55.95887 | 4.772295 |
|  | Bra023093 | BraA03g019280.3C.1 | 0.394552 | 0.425976 |  | 0 | 0 |
|  | Bra026597 | BraA02g035560.3C.1 | 0 | 0 |  | 0.150775 | 0 |
|  | Bra024482 | BraA06g029990.3C.1 | 0.646092 | 1.431524 |  | 0 | 0 |
|  | Bra015831 | BraA07g039130.3C.1 | 12.44743 | 14.11056 |  | 1.383304 | 1.16466 |
|  | Bra011892 | BraA06g044690.3C.1 | 13.97077 | 19.01782 |  | 18.09897 | 7.9319 |
|  | Bra034655 | BraA08g015700.3C.1 | 15.04516 | 13.26991 |  | 0 | 0 |
|  | Bra028841 | BraA02g000840.3C.1 | 0 | 0 |  | 0 | 0 |
|  | Bra024013 | BraA03g056500.3C.1 | 0 | 0 |  | 0 | 0 |
|  | Bra006438 | BraA03g008320.3C.1 | 13.88538 | 16.17206 |  | 26.15391 | 6.323237 |
|  | Bra023657 | BraA02g007440.3C.1 | 3.415396 | 3.363569 |  | 0 | 0 |
|  | Bra032322 | BraA09g035400.3C.1 | 0 | 0 |  | 0 | 0 |
|  | Bra032097 | BraA04g017940.3C.1 | 0 | 0 |  | 0.127057 | 0.117368 |
|  | Bra023663 | BraA02g007500.3C.1 | 6.884671 | 14.36651 |  | 64.853 | 5.374408 |
|  | Bra032317 | BraA09g035450.3C.1 | 1.538762 | 0.311611 |  | 1.178121 | 0.763675 |
|  | Bra017255 | BraA04g025760.3C.1 | 0 | 0 |  | 0 | 0 |
|  | Bra014476 | BraA04g001580.3C.1 | 30.27193 | 23.31443 |  | 0.555926 | 4.406723 |
|  | Bra031171 | BraA09g055720.3C.1 | 0 | 0 |  | 0 | 0 |
|  | Bra002120 | BraA10g022120.3C.1 | 6.986507 | 13.23799 |  | 45.31927 | 5.313078 |
|  | Bra011560 | BraA01g003540.3C.1 | 21.98608 | 22.94092 |  | 20.19052 | 2.385763 |
|  | Bra000497 | BraA03g024950.3C.1 | 91.87974 | 7.189286 |  | 2.69042 | 48.75613 |
|  | Bra014013 | BraA08g005980.3C.1 | 0 | 0 |  | 0 | 0 |
|  | Bra005223 | BraA05g008340.3C.1 | 0 | 0 |  | 0 | 0 |
|  | Bra035094 | BraA07g042150.3C.1 | 1.424874 | 0.28556 |  | 0 | 0 |
|  | Bra000380 | BraA03g023650.3C.1 | 8.053906 | 11.88827 |  | 0 | 0 |
|  | Bra026044 | BraA06g012490.3C.1 | 14.97181 | 12.63433 |  | 0.340275 | 1.884317 |
|  | Bra006577 | BraA03g009820.3C.1 | 3.520391 | 1.940918 |  | 0.987102 | 0.451981 |
|  | Bra006432 | BraA03g008260.3C.1 | 0.759636 | 0.275729 |  | 12.51469 | 1.16023 |
|  | Bra002130 | BraA10g022020.3C.1 | 9.371812 | 12.50435 |  | 21.51756 | 1.335297 |
|  | Bra000772 | BraA03g027900.3C.1 | 0 | 0 |  | 0 | 0.1383 |
|  | Bra037405 | BraA09g000950.3C.1 | 102.0096 | 97.49348 |  | 34.7125 | 84.82211 |
|  | Bra020841 | BraA08g015020.3C.1 | 0.336939 | 0.312817 |  | 2.358207 | 3.176778 |
|  | Bra010827 | BraA08g023850.3C.1 | 4.432578 | 6.443912 |  | 14.89571 | 1.381838 |
|  | Bra017747 | BraA03g059870.3C.1 | 0 | 0 |  | 0.853383 | 0 |
|  | Bra001981 | BraA07g004490.3C.1 | 0 | 0 |  | 0 | 0 |
|  | Bra039357 | BraA01g038490.3C.1 | 0.192954 | 0.076735 |  | 0.144404 | 0.141734 |
|  | Bra003044 | BraA03g014350.3C.1 | 2.167646 | 2.663732 |  | 1.41204 | 1.077123 |
|  | Bra023662 | BraA02g007490.3C.1 | 3.46543 | 1.59019 |  | 0 | 0 |
|  | Bra023656 | BraA02g007430.3C.1 | 3.953149 | 6.64061 |  | 0 | 0 |
|  | Bra007552 | BraA09g050440.3C.1 | 3.47874 | 1.646139 |  | 1.366228 | 1.363144 |
|  | Bra025770 | BraA06g015360.3C.1 | 0.23385 | 0.226849 |  | 0.085307 | 0.133584 |
|  | Bra001473 | BraA03g035520.3C.1 | 18.84201 | 11.67486 |  | 1.407429 | 5.307999 |
|  | Bra033581 | BraA06g044670.3C.1 | 27.80288 | 43.00023 |  | 18.56824 | 1.34321 |
|  | Bra022663 | BraA02g014480.3C.1 | 3.960379 | 4.939129 |  | 24.31969 | 23.66368 |
|  | Bra000682 | BraA03g026940.3C.1 | 0 | 0 |  | 0 | 0 |
|  | Bra006576 | BraA03g009810.3C.1 | 0 | 0 |  | 0 | 0 |
|  | Bra008999 | BraA10g027100.3C.1 | 0 | 0 |  | 0 | 0 |
|  | Bra011891 | BraA01g000130.3C.1 | 9.502669 | 9.872046 |  | 77.72805 | 29.11691 |
|  | Bra008032 | BraA02g020990.3C.1 | 0.684045 | 1.987204 |  | 66.45833 | 24.83911 |
|  | Bra029452 | BraA09g026350.3C.1 | 2.314029 | 2.072794 |  | 7.468019 | 3.918977 |
|  | Bra012188 | BraA06g015440.3C.1 | 4.022225 | 3.472982 |  | 0.289646 | 0.631338 |
|  | Bra006435 | BraA03g008290.3C.1 | 3.75399 | 6.969262 |  | 41.01771 | 4.873242 |
|  | Bra034656 | BraA08g015690.3C.1 | 10.55635 | 12.55127 |  | 1.027936 | 0.392992 |
|  | Bra009535 | BraA10g032690.3C.1 | 0 | 0.254921 |  | 0.84322 | 1.842997 |
|  | Bra002124 | BraA10g022080.3C.1 | 17.6934 | 22.18248 |  | 64.90862 | 8.629535 |
|  | Bra008209 | BraA02g022940.3C.1 | 16.36319 | 13.00636 |  | 4.377915 | 5.144347 |
|  | Bra003881 | BraA07g029140.3C.1 | 2.036758 | 2.549814 |  | 5.509604 | 12.05185 |
|  | Bra039264 | BraA04g032240.3C.1 | 40.61724 | 34.43498 |  | 1.62648 | 4.330086 |
|  | Bra026717 | BraA09g058230.3C.1 | 0 | 0 |  | 0 | 0.066114 |
|  | Bra020128 | BraA02g009140.3C.1 | 0 | 0 |  | 0 | 0 |
|  | Bra036830 | BraA09g042400.3C.1 | 0 | 0 |  | 0 | 0 |
|  | Bra023660 | BraA02g007470.3C.1 | 4.858843 | 6.087747 |  | 1.422461 | 0 |
|  | Bra011889 | BraA01g000150.3C.1 | 19.95718 | 36.06907 |  | 90.02588 | 20.60566 |
|  | Bra008207 | BraA02g022900.3C.1 | 0 | 0.043985 |  | 0.380394 | 2.240311 |
|  | Bra002128 | BraA10g022040.3C.1 | 9.194864 | 13.078 |  | 7.697402 | 0.491246 |
|  | Bra016606 | BraA08g029320.3C.1 | 0.16676 | 0.664326 |  | 0 | 0 |
|  | Bra016493 | BraA08g028080.3C.1 | 1.135977 | 0.386439 |  | 0 | 0.268101 |
|  | Bra034653 | BraA08g015710.3C.1 | 3.393725 | 2.001475 |  | 0 | 0 |
|  | Bra037243 | BraA07g002710.3C.1 | 0.90154 | 0.773077 |  | 0.738657 | 0.332782 |
|  | Bra002127 | BraA10g022050.3C.1 | 6.283255 | 10.60507 |  | 10.45767 | 1.056525 |
|  | Bra025774 | BraA06g015440.3C.1 | 4.022225 | 3.472982 |  | 0.289646 | 0.631338 |
|  | Bra023055 | BraA03g018910.3C.1 | 0 | 0 |  | 0 | 0 |
|  | Bra002131 | BraA10g022010.3C.1 | 3.270904 | 4.429379 |  | 38.25794 | 1.536638 |
|  | Bra011662 | BraA01g002510.3C.1 | 3.197662 | 2.973501 |  | 2.805841 | 0.601274 |
|  | Bra006436 | BraA03g008300.3C.1 | 2.075862 | 3.553235 |  | 5.551095 | 0 |
|  | Bra025981 | BraA06g013150.3C.1 | 0.385622 | 0 |  | 0.159709 | 0.417774 |
|  | Bra010826 | BraA08g023840.3C.1 | 13.61343 | 13.33722 |  | 34.13741 | 4.684041 |
|  | Bra010828 | BraA08g023860.3C.1 | 11.48071 | 15.88442 |  | 12.35812 | 2.177027 |
|  | Bra002330 | BraA10g019860.3C.1 | 0 | 0 |  | 0 | 0 |
|  | Bra018478 | BraA02g027890.3C.1 | 0 | 0 |  | 0 | 0 |
|  | Bra023655 | BraA02g007420.3C.1 | 0.121385 | 0.517484 |  | 0 | 0 |
|  | Bra017676 | BraA03g059020.3C.1 | 18.40962 | 24.57739 |  | 12.70345 | 0.480412 |
|  | Bra011559 | BraA01g003550.3C.1 | 2.798572 | 2.179276 |  | 6.948714 | 8.684085 |
|  | Bra023661 | BraA02g007480.3C.1 | 3.910327 | 6.04107 |  | 0 | 0 |
|  | Bra002123 | BraA10g022090.3C.1 | 5.251246 | 3.248694 |  | 13.39406 | 0.828372 |
|  | Bra011561 | BraA01g003530.3C.1 | 18.90034 | 11.82413 |  | 0 | 0.412511 |
